# Annexin A1 as a Key Modulator of Inflammatory, Glial, and Angiogenic Signaling Pathways in Diabetic Retinopathy

**DOI:** 10.1167/iovs.67.10.12

**Published:** 2026-08-05

**Authors:** Rafael André da Silva, Luiz Philipe de Souza Ferreira, Vinicius Moraes de Paiva Roda, Diego Dias dos Santos, Caroline Silvério Faria, Barbara Dalmaso, Giovanna Serra da Silva, Carolina Beltrame Del Debbio, Daniel Rodrigues de Bastos, Silvana Sandri, Sandra Helena Poliselli Farsky, Caio Vinicius Saito Regatieri, Cristiane Damas Gil

**Affiliations:** 1 Biosciences Graduate Program, Institute of Biosciences, Letters and Exact Sciences, UNESP, São José do Rio Preto, SP, Brazil; 2 Department of Morphology and Genetics, Escola Paulista de Medicina, Universidade Federal de São Paulo (EPM/UNIFESP), São Paulo, SP, Brazil; 3 Laboratory of Immunology, Heart Institute (InCor), Universidade de São Paulo, São Paulo, Brazil; 4 Laboratorio de Investigacao Medica (LIM03), Hospital das Clinicas HCFMUSP, Faculdade de Medicina, Universidade de Sao Paulo, SP, Brazil; 5 Department of Cell and Developmental Biology, Biomedical Sciences Institute, University of Sao Paulo, Sao Paulo, Brazil; 6 Facultad de Ciencias Médicas, Universidad Privada del Este filial Ciudad del Este, Paraguay; 7 Department of Clinical and Toxicological Analyses, USP, São Paulo, SP, Brazil; 8 Department of Ophthalmology, Escola Paulista de Medicina, Universidade Federal de São Paulo (EPM/UNIFESP), São Paulo, SP, Brazil

**Keywords:** Annexin A1, diabetic retinopathy, gliosis, inflammation, neovascularization

## Abstract

**Purpose:**

Annexin A1 (AnxA1) regulates inflammation and neovascularization in ocular diseases, influencing growth factor signaling and cytoskeletal dynamics. Accordingly, this study aimed to investigate the role of the AnxA1 protein in diabetic retinopathy (DR) and to understand how its absence affects inflammation and vascular dysfunction in DR.

**Methods:**

DR model was induced 12 weeks after streptozotocin injection in wild-type (AnxA1^+/+^) and AnxA1 knockout (AnxA1^−/−^) mice. We evaluated the consequences of AnxA1 deficiency on retinal morphology, Müller cell activation, inflammation, and angiogenesis associated with DR. We also performed an in silico analysis in wild-type animals with DR induced for 6 weeks and in a dataset of patients with DR. Furthermore, a choroidal neovascularization model was used to evaluate the role of AnxA1 in angiogenesis signaling.

**Results:**

AnxA1 deficiency exacerbated selective systemic inflammatory alterations and promoted retinal neurodegeneration associated with DR. AnxA1^−/−^ diabetic animals exhibited increased circulating IL-6, IL-17, chemokine (C-X-C motif) ligand 1, and monocyte chemoattractant protein-1 levels, retinal monocyte chemoattractant protein-1 and IL-4 upregulation, dysregulated MAPK/STAT3 signaling, and altered gliosis responses. In addition, AnxA1 deficiency was associated with a proangiogenic signaling profile characterized by increased retinal levels of EGF, VEGF-A, and endothelin-1 in DR, whereas the upregulation of VEGF-C was predominantly observed in the choroidal neovascularization model.

**Conclusions:**

Endogenous AnxA1 modulates inflammatory–glial and proangiogenic signaling pathways in the diabetic retina. Its deficiency is associated with selective inflammatory dysregulation and enhanced angiogenesis-related signaling during DR progression.

Diabetes mellitus is considered a global public health problem associated with multiple ocular complications, particularly diabetic retinopathy (DR), one of the leading causes of acquired blindness in the working-age population.^1^^,^^2^ DR is currently recognized as a complex neurovascular and inflammatory disease characterized by progressive microvascular dysfunction, retinal ischemia, neurodegeneration, and pathological neovascularization.^3^ DR progresses from nonproliferative DR, marked by microvascular abnormalities and increased vascular permeability, to proliferative DR, in which retinal ischemia triggers aberrant neovascularization and vitreous hemorrhage.^4^^,^^5^

Several factors contribute to the development and progression of DR, including hyperglycemia, oxidative stress, hypoxia, dyslipidemia, and chronic inflammation.^6^^,^^7^ Müller glial cells represent the major glial population in the retina and provide structural and metabolic support throughout the retinal layers.^8^ Under diabetic conditions, Müller cells become activated and undergo reactive gliosis, a process characterized by increased expression of glial fibrillary acidic protein (GFAP) and vimentin.^9^^,^^10^

Activated Müller cells contribute directly to retinal inflammation and vascular dysfunction through the production of cytokines, chemokines, nitric oxide, and angiogenic mediators, including VEGF, FGF, insulin-like growth factor-1, and TGF-β.^8^^,^^10^^–^^14^ Given their central role in inflammatory amplification and angiogenic signaling, Müller glial cells are considered critical mediators of DR progression.

In this context, Annexin A1 (AnxA1) emerges as a biologically relevant candidate in DR pathophysiology. AnxA1 is a calcium-dependent phospholipid-binding protein that interacts with formyl peptide receptors (FPRs), particularly FPR1 and FPR2, regulating leukocyte trafficking, cytokine production, endothelial activation, and resolution of inflammation.^15^^–^^18^ Beyond its well-established proresolving properties, AnxA1 has also been implicated in neuroprotection, intermediate filament regulation, fibrosis, and glial activation.^19^^,^^20^

AnxA1 is expressed in different cells of the retina, including neuronal cells, macroglia, and especially microglia.^21^ Evidence from experimental ocular disease models further supports the relevance of AnxA1 in retinal homeostasis and inflammatory regulation. In an animal model of infectious uveitis and in lipopolysaccharide-activated ARPE-19 cells, AnxA1 is overexpressed and indicated in the resolution of ocular inflammation, both in vivo and in vitro.^22^ In the in vitro model, AnxA1 and its peptide were also shown to negatively regulate the fibrosis-related growth factor.^23^ Autoimmune uveitis shows overexpression of AnxA1 in the human retina and mice, and treatment with AnxA1 reduces inflammation.^24^ In an in vitro glaucoma model, treatment with the AnxA1 peptide suppresses cell death in retinal ganglion cells.^25^ Collectively, these findings indicate that AnxA1 may act as an endogenous modulator of retinal inflammation, glial reactivity, and vascular dysfunction.

Current therapies for DR, including intravitreal anti-VEGF agents and corticosteroids, primarily target inflammation and neovascularization.^26^ Although these approaches have improved clinical outcomes, significant limitations remain, including incomplete therapeutic response, persistent gliosis, resistant inflammation, and adverse effects such as increased intraocular pressure and cardiovascular complications.^27^^–^^31^ These limitations reinforce the need to identify endogenous regulatory pathways capable of simultaneously modulating inflammatory and angiogenic mechanisms involved in DR progression.

Despite growing evidence supporting the anti-inflammatory and proresolving functions of AnxA1 in ocular diseases, its role in DR remains poorly understood. Therefore, this study investigated the impact of endogenous AnxA1 deficiency on retinal inflammation, Müller cell gliosis, vascular dysfunction, and angiogenic signaling in experimentally induced DR.

## Material and Methods

### Induction of DR

The experimental procedures were approved by UNIFESP's Internal Biosafety Committee and Ethics Committee on Animal Experimentation (CEUA No. 8518230821) and were conducted in accordance with the ARVO Statement for the Use of Animals in Ophthalmic and Vision Research. Two strains of mice were used: wild-type (AnxA1^+/+^) and knockout (AnxA1^−/−^) C57BL/6^32^^–^^34^ males aged between 10 and 12 weeks and weighing between 20 and 25*g* from the Center for the Development of Experimental Models for Medicine and Biology, UNIFESP. The animals were given water and food (ad libitum) in an appropriate room with soundproofing, controlled temperature (22°C ± 1°C), and a light/dark cycle (12:12 hours). Animals were randomly allocated to experimental groups, and all outcome analyses were performed in a masked fashion.

The streptozotocin (STZ)-induced diabetes model was used.^35^^,^^36^ STZ was diluted on the day of the experiment in 0.1 M sodium citrate buffer (pH 4.5), following the standard protocol recommended by the *Animal Models of Diabetic Complications Consortium* (http://www.diacomp.org/). The two strains of mice were divided into two groups: (1) controls (nondiabetic) received only injections of the vehicle (0.1 M sodium citrate buffer, pH 4.5); and (2) DR, challenged with STZ, 65 mg/kg, through five consecutive daily intraperitoneal injections. Experiments were conducted in two independent experimental sets containing six animals per group for each strain and condition.

Blood samples were collected from the tail vein to determine glucose levels using test strips and the Accutrend Plus meter (Roche, Basel, Switzerland) and also weighed monthly (30, 60, and 90 days). Only animals with consistently high blood glucose levels (>350 mg/dL) were considered diabetic and used in the study. After 90 days (12 weeks) of protocol induction, the animals were euthanized using an excessive dose of anesthetic—ketamine (350 mg/kg) and xylazine (37.5 mg/kg)—intraperitoneally.

Both eyes (right and left) were used for distinct experimental analyses; however, each retina was allocated to a single assay to avoid pseudoreplication. In the first experimental set, right eyes were processed for histological and immunohistochemical analyses, whereas left retinas were used for retinal homogenate preparation for Western blotting and multiplex assays. In the second experimental set, right retinas were processed for RT-qPCR analyses, and left retinas were used to complement multiplex assays. All tissue processing and quantitative analyses were performed in a masked fashion regarding genotype and experimental condition.

### Induction of Choroidal Neovascularization (CNV)

The laser-induced CNV mouse model was performed as previously described.^37^ Twelve-week-old AnxA1^+/+^ and AnxA1^−/−^ mice were used and divided into laser-induced CNV and control groups (*n* = 6/group). Laser photocoagulation was performed using a 532 nm argon laser (IRIDEX Oculight GLx, Mountain View, CA, USA) coupled to a slit lamp delivery system (Carl Zeiss 30SL-M, Jena, Germany). Four laser spots (spot size 50 µm, duration 100 ms, and power 150 mW) were applied at the 3, 6, 9, and 12 o'clock positions around the optic nerve head. The resulting bubble formation indicated the rupture of Bruch's membrane. Intraperitoneal anesthesia was administered with weight-adjusted doses of ketamine 100 mg/kg and xylazine 10 mg/kg. After 5 days of induction, the animals were euthanized for further analysis.

### Flow Cytometry

Flow cytometry experiments were carried out to characterize the expression of CD3 (T cells), Ly6G (neutrophils), and F4/80 (monocytes). Peripheral blood cells were obtained after red blood cell lysis with ammonium chloride and fixed with FACS lysing solution for 30 minutes at room temperature, washed with PBS containing 0.1 M glycine and incubated with FITC rat anti-mouse CD3 BD Pharmigen (1:50, cat.#555274, BD Pharmigen, Franklin Lakes, NJ, USA), FITC-conjugated anti-Ly6G (1:50; cat.#551461, BD Pharmigen), and PerCP/Cyanine5.5 anti-mouse F4/80 (1:400, cat.#123127, BioLegend, San Diego, CA, USA) for 40 minutes in the dark at room temperature. The cells were analyzed using a flow cytometer (BD Accuri C6), with 10,000 events analyzed and the BD CSampler Analysis software (version 1.0.2641–21, BD Biosciences, Franklin Lakes, NJ, USA) was used.

### Cytokine and Growth Factor Quantification

Levels of TNF-α, IL-1β, IL-2, IL-6, IL-10, IL-17, chemokine (C-X-C motif) ligand 1 (CXCL1), and monocyte chemoattractant protein-1 (MCP-1) were quantified using the multiplex cytokine panel (#MHSTCMAG-70KPMX; Millipore Corporation, Burlington, MA, USA). In addition, angiogenic factors including VEGF-A, VEGF-C, VEGF-D, endothelin-1, EGF, and FGF-2 were measured using the angiogenesis panel (#MAGPMAG-24K) and the TGF-β magnetic bead kit (#TGFBMAG-64K; TGF-β1, TGF-β2, and TGF-β3). For each assay, 25 µL of plasma or retinal homogenate was used (protein extraction and quantification described elsewhere in this article). Analyte detection was performed according to the manufacturer's instructions using MAGPIX system (xMAP technology by Luminex, Austin, TX, USA). Results are expressed as mean ± standard error of the mean (SEM) concentrations of the different targets in pg/mL (plasma) or picograms per milligram of protein (retinal homogenate).

### Western Blot

After euthanasia, retinas were dissected and immediately stored at −80°C. Proteins were extracted by homogenization using a BeadBlaster 24 homogenizer (Benchmark Scientific, Sayreville, NJ, USA) in RIPA lysis buffer (#89900, Thermo Fisher Scientific, Waltham, MA, USA) supplemented with protease (#A32963, Thermo Fisher Scientific) and phosphatase inhibitor (#49068450001, Roche). Homogenates were centrifuged at 12,000 rpm for 10 minutes at 4°C, and the supernatants were collected. This procedure was repeated twice to ensure complete elimination of any cell debris. Protein concentrations were determined using the Qubit system (#Q33210, Quant-iT Protein Assay Kit, Invitrogen, Thermo Fisher Scientific). Samples were diluted 1:1 v/v in Laemmli buffer (#1610737, Bio-Rad Laboratories, Hercules, CA, USA) and heated in a dry bath at 95°C for 5 minutes. Protein samples (30 µg/well) were loaded onto SDS-PAGE at 12% or 15% with the molecular weight markers (#26616, Thermo Fisher Scientific) and subsequently transferred to 0.45-µm nitrocellulose membranes (#1620115, Bio-Rad Laboratories). The nitrocellulose membranes were blocked with skim milk or BSA (5%) in Tris-buffered saline and 0.1% tween 20 (TTBS) for 2 hours and incubated overnight at 4°C with antibodies (Table 1) diluted in TTBS with 1% skim milk or BSA.

**Table 1.** List of Primary Antibodies Used in Western Blotting Analysis

Antibodies	Reference	Dilution
Rabbit anti-**AnxA1**	#713400 Invitrogen	1:1000
Rabbit anti-FPR2	#720367 Invitrogen	1:1000
Mouse anti-phospho-p44/42 MAPK (Erk1/2) (Thr202/Tyr204) (E10)	#9106 Cell Signaling	1:1000
Rabbit anti-p44/42 MAP kinase (Erk1/Erk2)	#9102l Cell Signaling	1:1000
Mouse anti-GFAP	#G3893 Sigma-Aldrich	1:2000
Rabbit anti-vimentin	#5741 Cell Signaling	1:1000
Rabbit anti-Stat3	#A11216 Abclonal	1:1000
Rabbit anti-phospho.pStat3	#AP0474 Abclonal	1:1000
Rabbit anti-blutamine synthetase	#G2781 Sigma-Aldrich	1:2000
Mouse anti-phospho-SAPK/JNK (Thr183/Tyr185) (G9)	#9255 Cell Signaling	1:1000
Rabbit anti-SAPK/JNK	#9252 Cell Signaling	1:1000
Rabbit anti-p38	#9212L Cell Signaling	1:1000
Mouse anti-phospho-p38	#9216L Cell Signaling	1:1000
Rabbit anti-glyceraldehyde-3-phosphate dehydrogenase (GAPDH)	#G9545 Sigma-Aldrich	1:3000

GAPDH was used as an endogenous control. After washing in TTBS, the membranes were incubated at room temperature for 2 hours with specific secondary antibodies (1:3000; Thermo Fisher Scientific Inc, Rockford, MI, USA), washed in TTBS (3 × 10 minutes) and revealed by chemiluminescent amplification (SuperSignal West Pico Chemiluminescent Substrate, Thermo Fisher Scientific #34578). Protein expression was analyzed by densitometry (arbitrary units) using ImageJ 1.53k software (National Institutes of Health) to determine relative expression concerning the endogenous control, and then the fold change was calculated.

### Histomorphometric Analysis

Eyes were fixed in 4% paraformaldehyde (#30525-89-4, Sigma-Aldrich, St. Louis, MO, USA) for 24 hours, washed in tap water, dehydrated in a graded ethanol series, and embedded in paraffin. Sections (4 µm) were obtained using a Leica RM2155 microtome (Leica Microsystems, Nussloch, Germany) and stained with hematoxylin and eosin for retinal morphometric analysis. Slides were scanned using an Aperio CS2 slide scanner (Leica Microsystems, Wetzlar, Germany). Total retinal thickness was measured using QuPath software (version 0.5.1; University of Edinburgh, Edinburgh, UK). Three perpendicular lines were taken in the peripheral nasal and temporal regions of the retina and the central region, and the average of the three measurements of each region was taken for later analysis. QuPath was also used to quantify the total number of cells in the outer nuclear layer (ONL) and inner nuclear layer (INL). Retinal thickness values (µm) and cell densities (cells/mm²) are presented as mean ± SEM.

### RT-qPCR

Total RNA from retinal tissue was extracted using TRIzol reagent, and first-strand cDNA was synthesized with RevertAid First Strand cDNA Synthesis Kit following the manufacturer's instructions (Thermo Fisher Scientific). Quantitative PCR was performed using the QuantiFast SYBR Green PCR kit (Qiagen, Hilden, Germany) and gene-specific primers for mouse samples as listed in Table 2. The housekeeping gene 18s rRNA (mouse) was used as the endogenous control. Amplification and fluorescence detection were carried out on an Applied Biosystems StepOnePlus Real-Time PCR System (Thermo Fisher Scientific). Relative mRNA expression levels were calculated using the comparative threshold cycle (Ct) and expressed relative to the controls (ΔΔCt method).^38^ All reactions were run in duplicate.

**Table 2.** Primer Sequences Used for qPCR Analyses

Target Gene	Forward Primer (5′→3′)	Reverse Primer (5′→3′)
***Rho***	TGCCACACTTGGAGGTGAAA	ACCACGTAGCGCTCAATGG
***OPN1***	ACTCAGCATCATCGTGCTCTGCTA	AGTATGCGAAGACCATCACCACCA
***GFAP***	GCAAAAGCACCAAAGAAGGGGA	ACATGGTTCAGTCCCTTAGAGG
***Vim***	CAACCGGAGCTATGTGACCA	CGAGAAGTCCACCGAGTCTT
***SYP***	ACTTCAGGACTCAACACCTCGG	GAACCATAGGTTGCCAACCCAG
**18s rRNA**	GGACAGGATTGACAGATTGATAGC	TGCCAGAGTCTCGTTCGTTA

Forward and reverse primer sequences for photoreceptor markers of rods and cones -Rhodopsin (*Rho*) and medium-wavelength Opsin (*Opn1*) - as well as for glial activation markers, including glial fibrillary acidic protein (*Gfap)* and vimentin (*Vim*), and the neuronal synaptic marker Synaptophysin (*Syp*), are provided for mouse, rat, and human samples, as applicable. The housekeeping gene 18s rRNA was used as the internal control for mouse samples.

### Immunohistochemistry

After histological processing, tissue sections were subjected to antigen retrieval using citrate buffer (10 mM, pH 6.0), followed by blocking of endogenous peroxidase activity. Sections were then incubated overnight at 4°C with anti-GFAP primary antibody (1:500; #G3893, Sigma-Aldrich). As a negative control, the sections were incubated without the primary antibody. The sections were then washed in PBS and incubated with the peroxidase-conjugated secondary antibody (Sigma-Aldrich, USA) and revealed with 3,3'- diaminobenzidine. After this reaction, the slides were counterstained with Carazzi's hematoxylin. Coverslips were mounted, and images were acquired using a CCD camera coupled to a Zeiss Axioskop 2 microscope (Carl Zeiss) with a 10× objective under identical acquisition settings for all experimental groups. Immunohistochemistry was performed to qualitatively evaluate the distribution and localization pattern of GFAP immunoreactivity in retinal layers and Müller cell processes. Quantitative analysis of GFAP protein expression was performed separately by Western blotting using retinal homogenates.

### In Silico Analysis

A publicly available transcriptomic dataset was selected from the Gene Expression Omnibus (GEO) repository: GSE111465 (https://www.ncbi.nlm.nih.gov/geo/query/acc.cgi?acc=GSE111465), in which C57BL/6 mice were subjected to STZ-induced DR for 6 weeks, and control animals received no intervention. This dataset was used as complementary in silico evidence of early DR-associated modulation of the AnxA1/FPR2 axis. The datasets were analyzed individually using the GEO2R tool (http://www.ncbi.nlm.nih.gov/geo/geo2r/, accessed May 3, 2023), which enables differential gene expression analysis between experimental groups. GEO2R analysis was applied to evaluate *Anxa1* and *Fpr2* expression levels.

To further investigate the relevance of AnxA1 and gliosis-associated markers in human DR, the transcriptomic dataset GSE160306 (https://www.ncbi.nlm.nih.gov/geo/query/acc.cgi?acc=GSE160306; GPL20301 Illumina HiSeq 4000, Homo sapiens; accessed May 3, 2023) was analyzed. This dataset comprises postmortem human retinal samples obtained from diabetic donors representing different stages of DR. For regional analysis, 29 retinal samples from the macular region and 30 from the retinal periphery were evaluated. In addition, samples were stratified according to Early Treatment Diabetic Retinopathy Study (ETDRS) severity scores as follows: 0 to 10 (*n* = 20), 20 to 35 (*n* = 20), 43 to 53 (*n* = 14), and 65 (*n* = 5). Normalized mRNA expression values (log2 counts per million) for *ANXA1* and *GFAP* were obtained and analyzed.

### Statistical Analysis

Data distribution was assessed using the Kolmogorov–Smirnov normality test. Statistical analyses were performed using GraphPad Prism software (v9.1). Parametric data were analyzed using two-way ANOVA followed by Tukey's multiple-comparison test or an unpaired Student *t*-test, as appropriate. Nonparametric data were analyzed using the Mann–Whitney or Kruskal–Wallis tests followed by Dunn's post hoc test. Outlier identification was performed using the ROUT method available in GraphPad Prism, and exclusions were applied only in cases of technical or biological inconsistency. Exact sample sizes for each analysis are indicated in the corresponding figure legends. Only one retina per animal was used for each experimental analysis. Variations in *n* values among experiments reflect sample availability and/or technical limitations. Data are presented as mean ± SEM, and differences were considered statistically significant at a *P* value of less than 0.05.

## Results

### Characterization of the Diabetes Model

**Figure 1.**
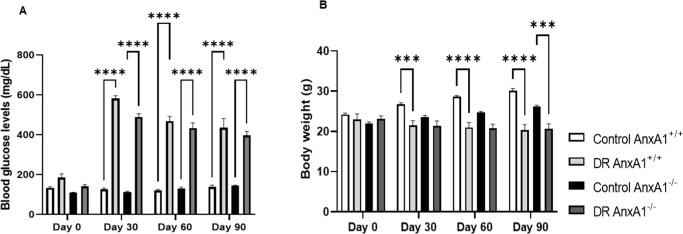
**Clinical parameters after diabetes induction.** (**A**) Blood glucose levels and (**B**) body weight. Data represent the mean ± SEM (*n* = 6 mice/group). Statistical analysis was performed using a two-way ANOVA, followed by Tukey's multiple-comparison test. ***P < 0.001; ****P < 0.0001.

STZ-induced diabetes recapitulates key features of type 1 diabetes leading to DR, with sustained hyperglycemia and body weight loss as hallmark characteristics of this model.^35^^,^^36^^,^^39^ Blood glucose levels showed that both AnxA1*^+/+^* and AnxA1^−/−^ mice exhibited a progressive and sustained increase in glycemia at 30, 60, and 90 days (****P < 0.0001) compared with their respective controls (Fig. 1A).

Body weight was recorded at 30, 60, and 90 days (Fig. 1B). AnxA1^+/+^ animals exhibited significant weight loss at all evaluated time points (***P < 0.001 and ****P < 0.0001). In contrast, AnxA1^−/−^ animals showed a tendency to lose weight at 30 and 60 days, with significant weight loss only observed at 90 days in comparison with the controls (***P < 0.001).

### Lack of Endogenous AnxA1 Exacerbates Systemic Inflammation in the DR Model

**Figure 2.**
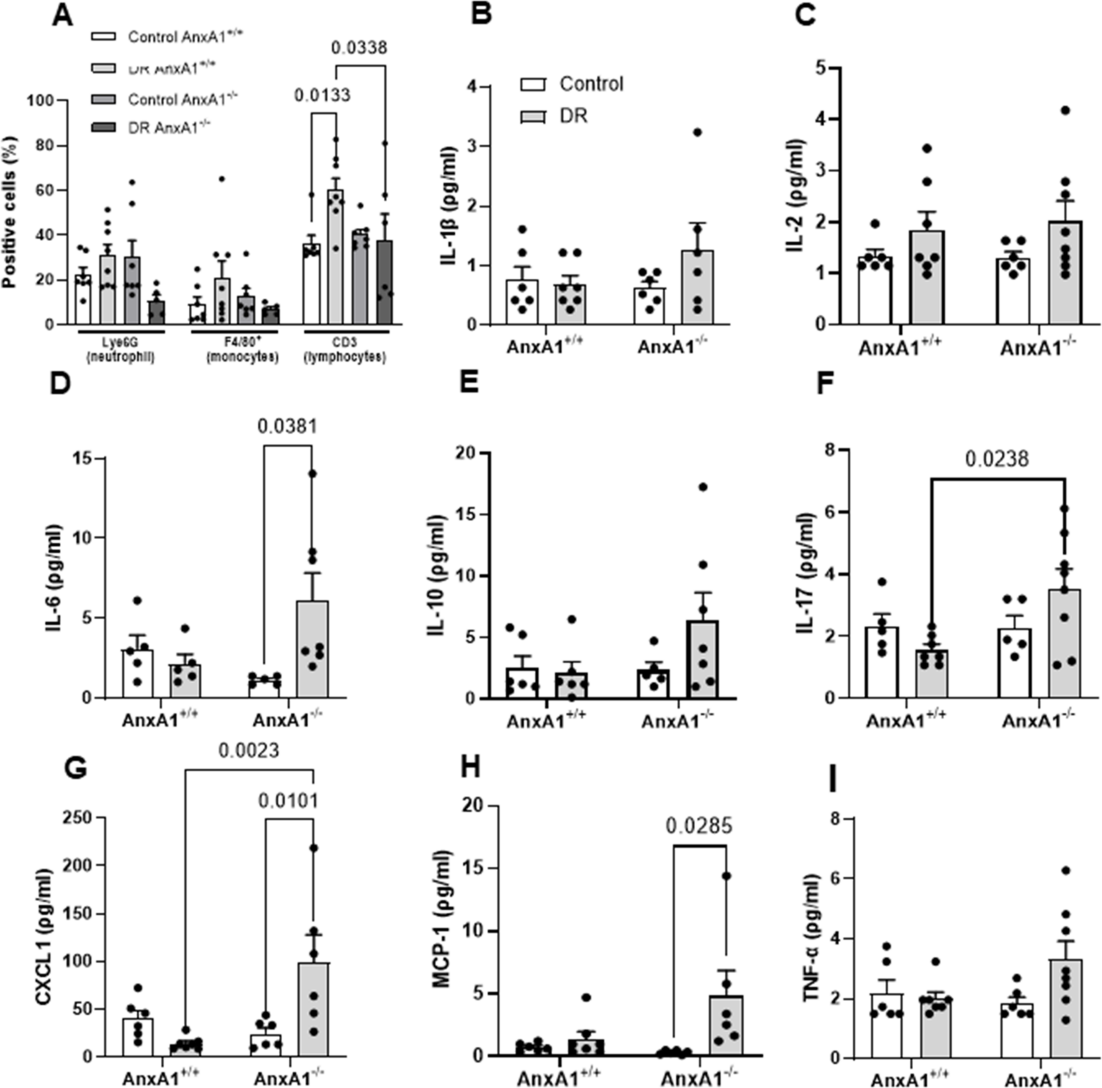
**AnxA1^−/−^ animals show exacerbated systemic inflammation.** (**A**) The proportion of neutrophils (Ly6G), monocytes (F4/80^+^), and lymphocytes (CD3) in the peripheral blood. Plasma levels of the interleukins (**B**) IL-1β, (**C**) IL-2, (**D**) IL-6, (**E**) IL-10, (**F**) IL-17, (**G**) CXCL1, (**H**) MCP-1, and (**I**) TNF-α. Data are presented as mean ± SEM of leukocyte proportion (%) or protein concentration (pg/mL), with dots representing individual animals (*n* = 6–8 animals/group). Statistical analysis was carried out using two-way ANOVA, followed by Tukey's multiple-comparison test.

Chronic low-grade inflammation arises due to prolonged hyperglycemia, oxidative stress, and other molecular mediators.^40^^,^^41^ To evaluate the leukocyte profile under the experimental conditions, flow cytometry was performed on peripheral blood leukocytes labeled with Ly6G (neutrophils), F4/80 (monocytes), and CD3 (T cells). At 90 days after the last dose of STZ (12 weeks), a significant increase in circulating T cells was detected in AnxA1^+/+^ animals with DR compared with both their respective controls and AnxA1^−/−^ animals with DR (*P < 0.05) (Fig. 2A). No significant changes were observed in neutrophil or monocyte populations among the experimental groups (Fig. 2A).

Analysis of interleukins and chemokines showed an increased plasma levels of IL-6, IL-17, CXCL1, and MCP-1 (Figs. 2D, 2F–H), only in AnxA1^−/−^ animals with DR compared with their control (*P < 0.05) and in relation to AnxA1^+/+^ animals with DR (**P < 0.01). IL-1β, IL-2, IL-10, CXCL1, and TNF-α showed no changes in any of the experimental conditions (Figs. 2B, 2C, 2E, 2I). These results show that the endogenous lack of AnxA1 exacerbates the systemic inflammatory response in DR.

### AnxA1 Deficiency Aggravates Retinal Structural Alterations in DR

**Figure 3.**
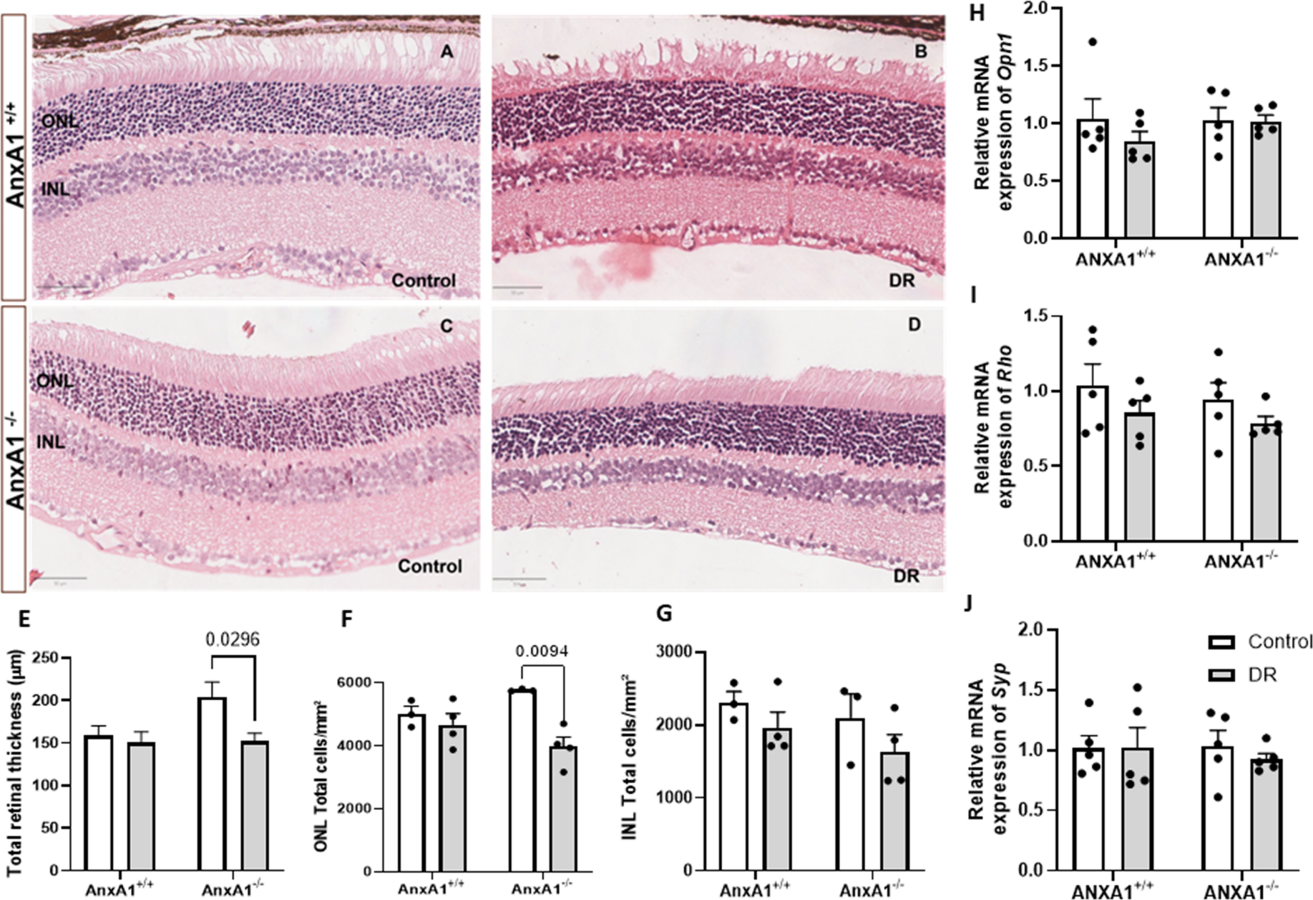
**Rod-specific retinal degeneration occurs in both strains.** Evaluation of total retinal thickness and number of total cells in the INL and ONL. (**A**–**D**) Representative photomicrographs of retinal sections from AnxA1^+/+^ and AnxA1^+/+^ mice under control and DR conditions. (**E**) Total retinal thickness, determined from three retinal fields per section for each animal, (**F**) total number of cells in the ONL, and (**G**) total number of cells in the INL. (**H**) Gene expression of *Opn1*, (**I**) *Rho*, and (**J**) *Syp.* The housekeeping gene 18s rRNA was used as the internal control for mouse samples. Data are presented as mean ± SEM, with dots representing individual animals (*n* = 3–5 animals/group). Retinal thickness is expressed in µm and cell quantification as total number of cells per square millimeter. Statistical analysis was carried out using two-way ANOVA, followed by Tukey's multiple comparison test. Representative hematoxylin and eosin (H&E)-stained images. Original magnification: 40×.

In the pathogenesis of DR, neuronal cells die, leading to a reduction mainly in photoreceptors located in the ONL,^42^ thereby contributing to retinal degeneration and progressive vision loss.^43^ After characterizing the systemic inflammatory profile in diabetic animals from both strains, retinal morphology was evaluated (Figs. 3A–G). Total retinal thickness and the number of cells in the INL and ONL were assessed. Quantitative analysis demonstrated a significant reduction in total retinal thickness and in the number of ONL cells in AnxA1^−/−^ mice with DR compared with their respective control group (Figs. 3E, 3F; **P < 0.01). No significant differences were observed in INL cell counts (Fig. 3G). To further investigate photoreceptor and synaptic integrity, the expression of *Rho* (rod photoreceptors), *Opn1* (cone photoreceptors), and *Syp* (synaptic marker) was analyzed (Figs. 3H–J). No significant alterations in the expression of the analyzed genes were detected among the experimental groups (Figs. 3H–J).

### Retinal Gliosis Is Attenuated in AnxA1-Deficient Mice During DR

**Figure 4.**
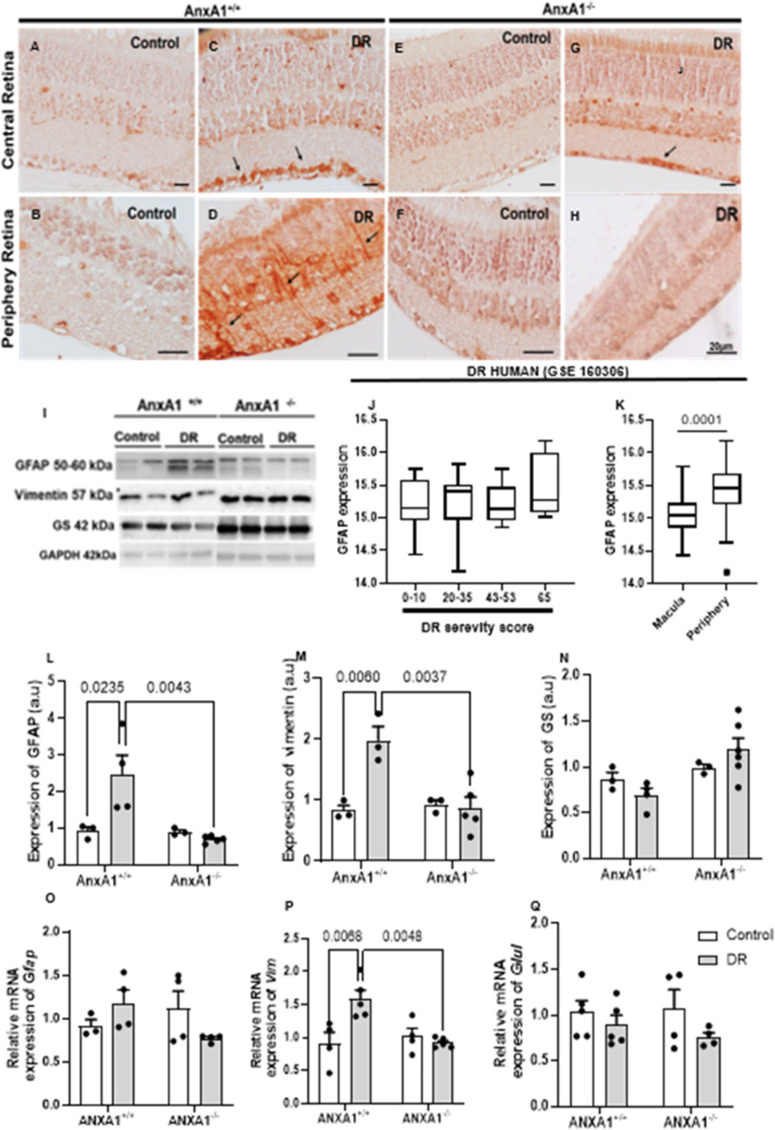
**AnxA1 regulates retinal gliosis in the DR model.** (**A**–**H**) Immunohistochemistry for the detection of GFAP in central and peripheral retina. AnxA1^+/+^ animals with DR exhibit gliosis (*arrows*), evidenced in the Müller cell layer (**C**, **D**). *Scale bars*, 20 µm. (**I**) Representative immunoblots of protein expression of (**L**) GFAP, (**M**) vimentin, (**N**) GS, and GAPDH (endogenous control). (**O**–**Q**) *Gfap*, *Vim*, and *Glul* gene expression. Data represent the mean ± SEM of protein (fold change) or gene expression, with dots representing individual animals (*n* = 3–5 animals/group). (**J**) Expression of *GFAP* transcripts according to ETDRS severity scores in patients with DR (GSE160306; ETDRS 0–10, *n* = 20; 20–35, *n* = 20; 43–53, *n* = 14; 65, *n* = 5). (**K**) Expression of *GFAP* transcripts in peripheral and macular retinas of patients with DR (GSE160306; *n* = 29 macula and 30 periphery). Statistical analysis was carried out using two-way ANOVA, followed by Tukey's multiple comparison test (**L**, **M**, **P**), or Mann–Whitney test (**K**).

Another key process in the pathogenesis of DR is retinal gliosis, in which Müller glial cells undergo hypertrophy and proliferation. These cells are the main source of proinflammatory and angiogenic mediators that lead to blindness.^44^ Thus, we initially evaluated the expression of the GFAP protein, the main marker of gliosis in Müller cells.^10^^,^^14^ Immunohistochemical analysis revealed markedly increased GFAP immunoreactivity in the inner limiting membrane of the central retina in AnxA1^+/+^ diabetic animals, consistent with activation at the Müller cell end feet (Figs. 4A, 4C). In the peripheral retina, AnxA1^+/+^ animals with DR also exhibited pronounced radial gliosis, evidenced by strong GFAP staining along Müller cell processes (Figs. 4B, 4D). In contrast, AnxA1^−/−^ retinas showed no appreciable differences in GFAP expression between the DR and control groups (Figs. 4E–H).

To corroborate these observations, we next assessed GFAP protein and gene expression levels in the retina. Consistent with the immunohistochemical findings, GFAP protein expression was significantly upregulated in AnxA1^+/+^ diabetic retinas relative to both their controls (*P* < 0.05) and AnxA1^−/−^ diabetic animals (*P* < 0.01) (Fig. 4L). In contrast, *Gfap* gene expression showed no alterations under different experimental conditions (Fig. 4O).

The analysis of vimentin, another established marker of Müller cell gliosis,^45^^,^^46^ revealed significantly elevated protein levels in AnxA1^+/+^ diabetic retinas compared with both their controls and AnxA1^−/−^ DR animals (*P* < 0.01) (Fig. 4M). *Vim* gene expression exhibited a similar profile (Fig. 4P). No significant changes in GS protein and *Glul* gene expression were detected among the experimental groups (Figs. 4N, 4Q).

To further support our findings, we performed an in silico analysis of human retinal transcriptomic datasets from the GSE160306 study (Figs. 4J–K). No changes in *GFAP* gene expression were observed across different DR severity scores (Fig. 4Q). However, *GFAP* transcript levels were significantly higher in the peripheral retina compared with the macula in patients with DR (****P < 0.0001) (Fig. 4K).

### Regulation of the AnxA1/FPR2 Axis in the Retina During DR

**Figure 5.**
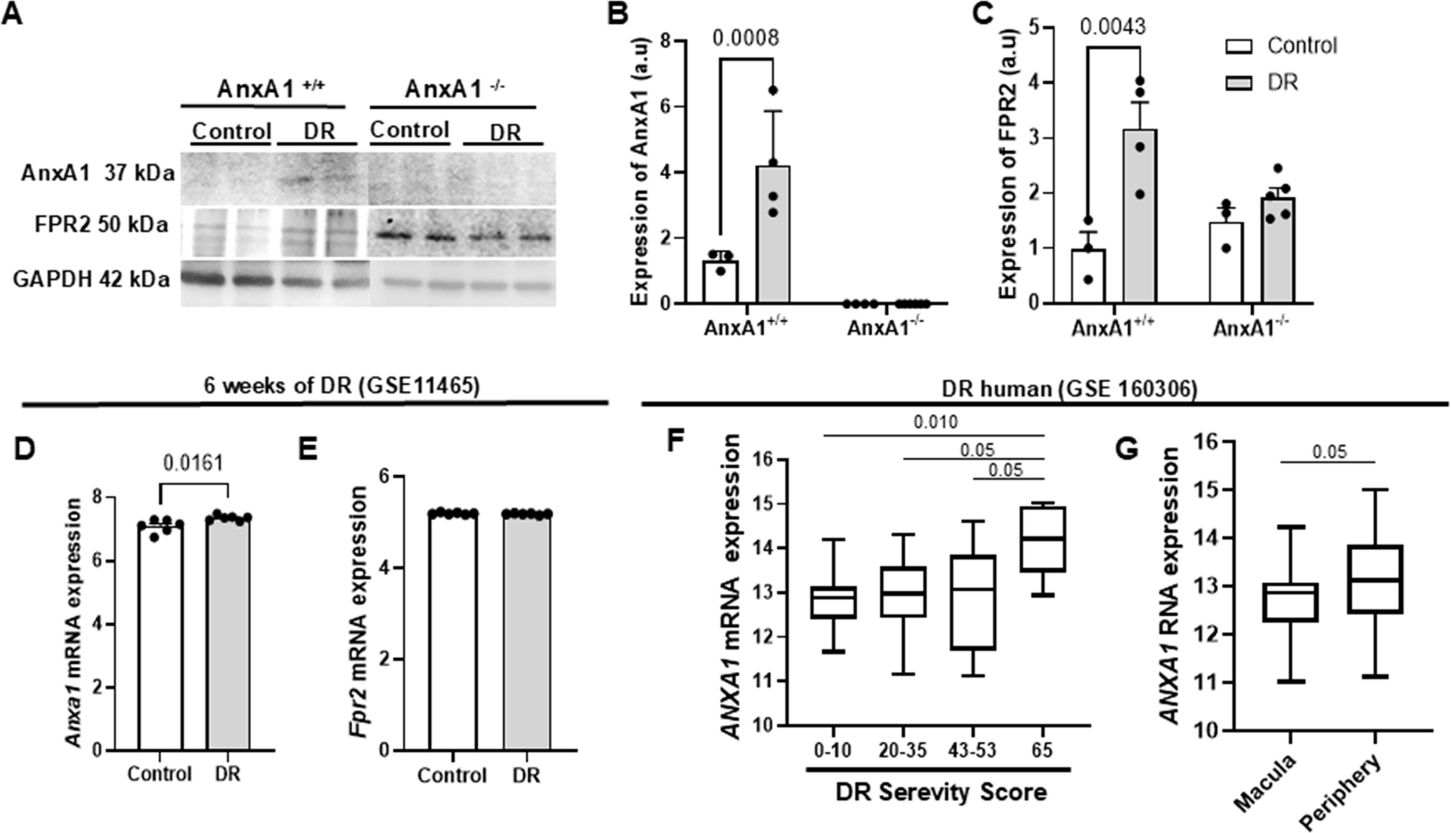
**Expression of AnxA1 and FPR2 in the pathogenesis of DR.** (**A**–**C**) Protein expression of AnxA1, FPR2, normalized by GAPDH (*n* = 3–5 animals/group). (**D**–**E**) Gene expression of *Anxa1* and *Fpr2* in the retina of animals 6 weeks after induction of DR (GSE111465; *n* = 6 mice/group). (**F**) Gene expression of *ANXA1* in the retina of humans, according to the severity of DR (GSE160306; ETDRS 0–10, *n* = 20; 20–35, *n* = 20; 43–53, *n* = 14; 65, *n* = 5). (**G**) Gene expression of *ANXA1* segregated by macular and peripheral region of the human retina (GSE160306; *n* = 29 macula and 30 periphery). Data are shown as mean ± SEM, with dots representing individual samples. Statistical analysis for the Western blot was performed using two-way ANOVA followed by Tukey's multiple comparison test (**B**, **C**). For GSE111465 and GSE160306, the Student *t*-test (**D**), Mann–Whitney test (**G**), and Kruskal–Wallis test followed by Dunn's test (**F**) were applied.

The AnxA1 protein and FPR2 has been implicated in various ocular pathologies, including glaucoma, uveitis, corneal injury, and conjunctivitis.^20^^,^^47^ To characterize the expression pattern of AnxA1, we evaluated its levels in murine retinal homogenates from wild-type DR and control groups using Western blotting. A significant upregulation of AnxA1 was detected in the retinas of the DR group compared with the control group (***P < 0.001) (Figs. 5A–B). Additionally, the absence of AnxA1 protein in knockout animals was confirmed (Figs. 5A–B). The expression of FPR2, the receptor for AnxA1, was also significantly elevated in the ANXA1^+/+^ retinas of the DR group compared with controls (**P < 0.01) (Figs. 5A, 5C). No changes in FPR2 levels were observed in AnxA1^−/−^ retinas (Figs. 5A, 5C).

To corroborate these findings, we performed an in silico analysis (GSE11465) of *Anxa1* and *Fpr2* mRNA expression in a murine model of STZ-induced DR over 6 weeks. Despite the difference in the induction period (6 weeks vs. our 12 weeks), a marked increase in *Anxa1* transcript levels was observed in the retinas of DR animals (Fig. 5D), while no significant changes were detected in *Fpr2* expression (Fig. 5E). To explore ANXA1 expression in humans, the GSE160306 dataset was analyzed. The data revealed differential *ANXA1* transcript expression across ETDRS severity groups in human retinas (Fig. 5F). Specifically, higher *ANXA1* expression was observed in samples classified as ETDRS 65 compared with ETDRS 0 to 10, 20 to 35, and 43 to 53 (*P < 0.05 and **P < 0.01, respectively). Moreover, *ANXA1* transcript levels were significantly elevated in the peripheral retina compared with the macula (*P < 0.05) (Fig. 5G).

### Loss of AnxA1 Selectively Enhances Inflammatory Mediators and Exacerbates Angiogenic Signaling in DR

**Figure 6.**
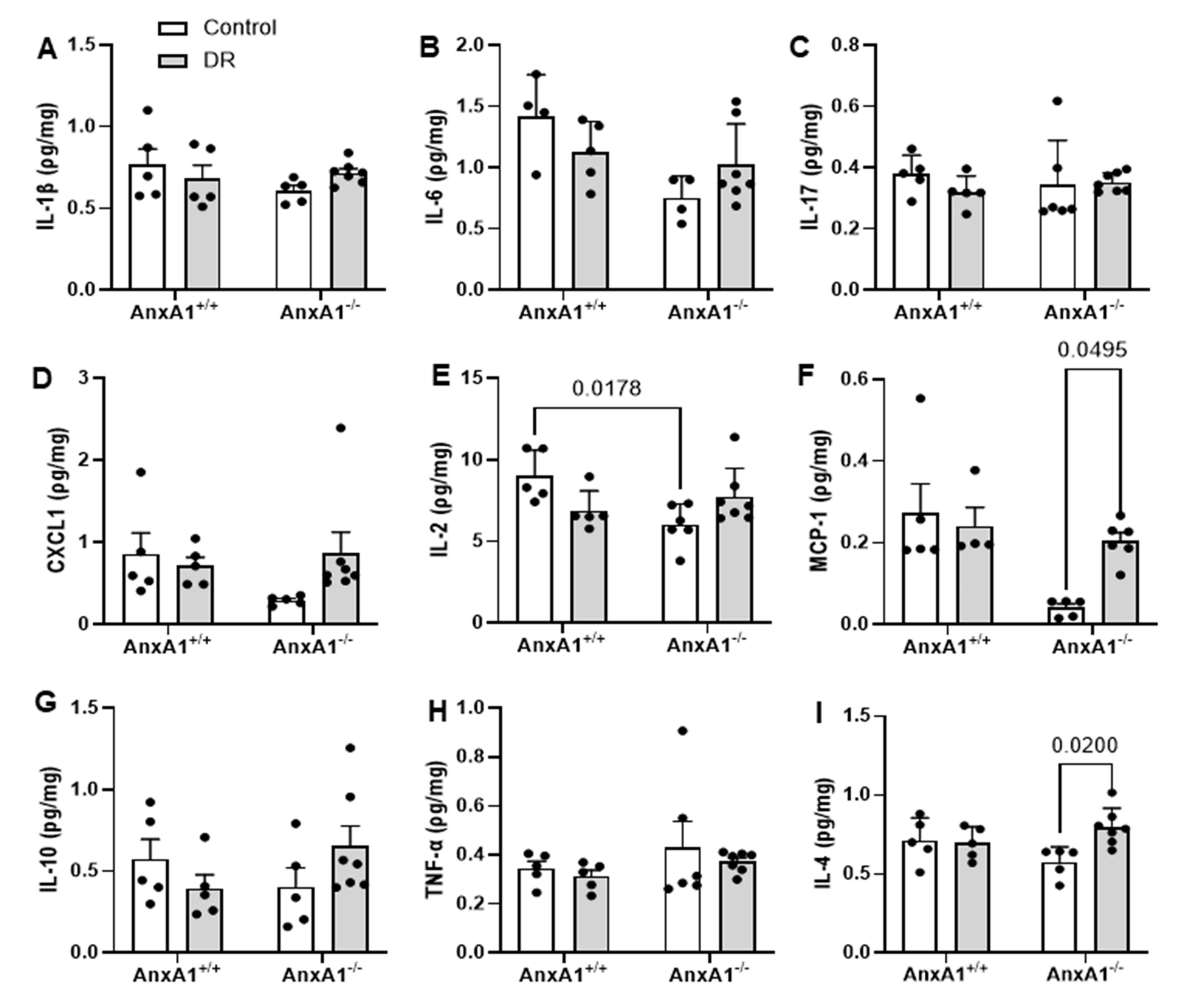
**Cytokine and chemokine levels in the retina in DR.** Retinal homogenates were analyzed for cytokine and chemokine levels: (**A**) IL-1β, (**B**) IL-6, (**C**) IL-17, (**D**) CXCL1, (**E**) IL-2, (**F**) MCP-1, (**G**) IL-10, (**H**) TNF-α, and (**I**) IL-4. Data are expressed as mean ± SEM, with dots representing individual animals (*n* = 4–7 animals/group). Statistical analysis was carried out using two-way ANOVA followed by Tukey's post hoc test.

To assess the impact of AnxA1 loss on inflammatory signaling in DR, we quantified cytokine and chemokine levels. This analysis revealed a significant increase in MCP-1 and IL-4 in the retinas of AnxA1^−/−^ mice with DR compared with controls (*P* < 0.05) (Figs. 6F, 6I). No significant changes were observed in IL-1β, IL-6, IL-17, CXCL1, IL-2, TNF-α, or IL-10 levels (Figs. 6A–E, 6G, 6H).

**Figure 7.**
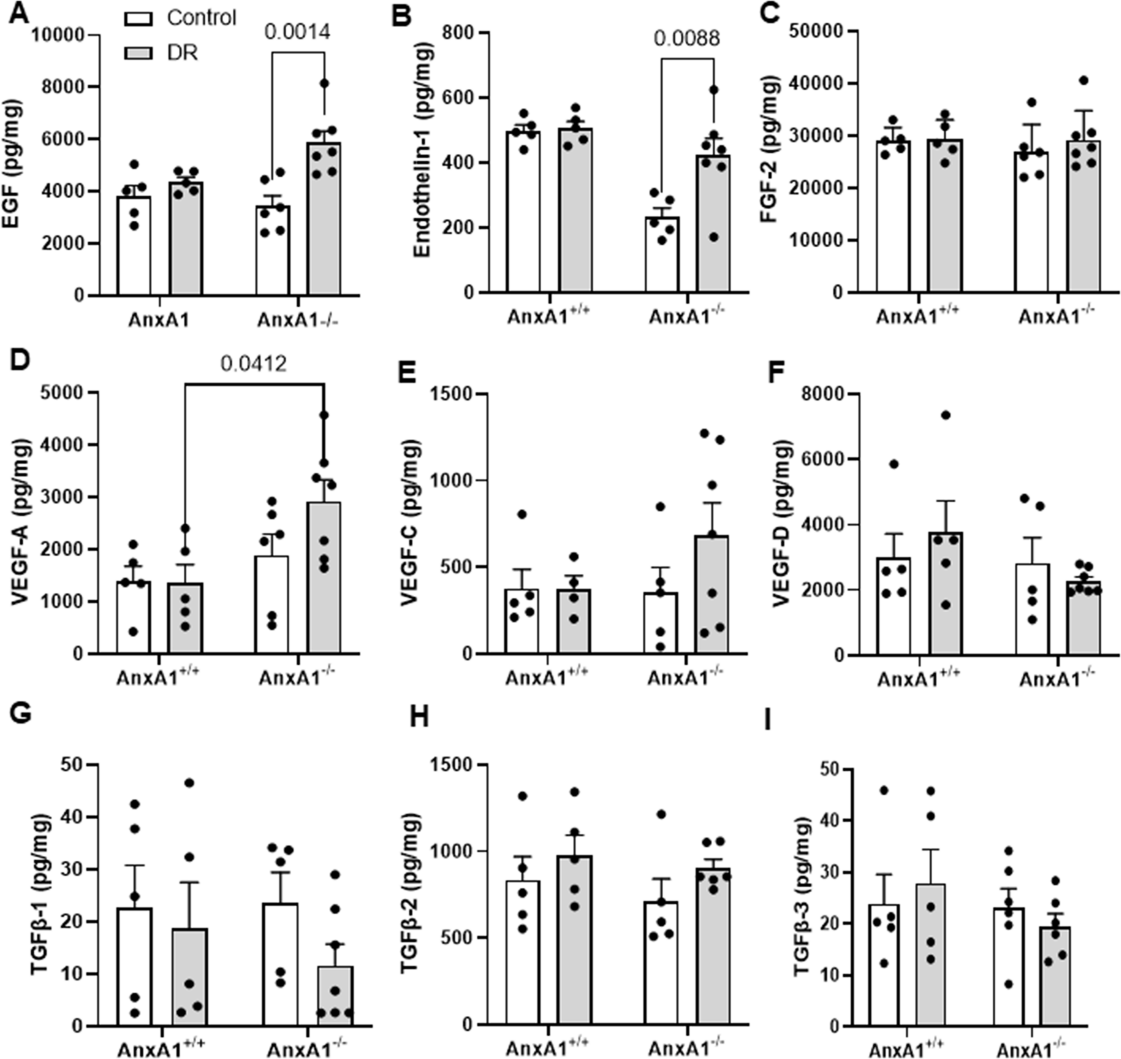
**Retinal levels of growth and angiogenic factors in a DR model.** Retinal homogenates were analyzed for (**A**) EGF, (**B**) endothelin-1, (**C**) FGF-2, (**D**) VEGF-A, (**E**) VEGF-C, (**F**) VEGF-D, (**G**) TGF-β1, (**H**) TGF-β2, and (**I**) TGF-β3. Data are expressed as mean ± SEM, with dots representing individual animals (*n* = 4–7 animals/group). Statistical analysis was carried out using two-way ANOVA followed by Tukey's post hoc test.

Given the established contribution of angiogenesis-associated mediators and vascular dysfunction to DR progression, we next assessed whether AnxA1 deficiency impacts these pathways. AnxA1^−/−^ mice with DR exhibit a significant increase in the retinal levels of EGF and endothelin-1 compared with their controls (**P < 0.001) (Figs. 7A, 7B). Additionally, VEGF-A levels were significantly higher in AnxA1^−/−^ animals with DR compared with AnxA1^+/+^ animals with DR (*P < 0.05) (Fig. 7D). In contrast, no significant alterations were detected in FGF-2, VEGF-D, or TGF-β isoforms levels (Figs. 7C, 7E–I).

To further investigate the role of AnxA1 in angiogenesis-associated signaling, a CNV model was used as a complementary experimental paradigm of pathological neovascularization. In this context, no significant differences were observed in the levels of inflammatory cytokines and chemokines, including IL-1β, IL-6, IL-17, CXCL1, IL-2, MCP-1, IL-10, TNF-α, or IL-4, under the evaluated conditions (Supplementary Fig. S1).

In contrast, analysis of growth and angiogenic factors revealed that AnxA1^−/−^ animals subjected to CNV exhibited significantly elevated levels of VEGF-C compared with both their respective controls and CNV-induced AnxA1^+/+^ animals (*P < 0.05; **P < 0.01; Supplementary Fig. S2). No significant changes were detected in EGF, endothelin-1, FGF-2, VEGF-A, VEGF-D, or TGF-β2 levels. Regarding the TGF-β family, TGF-β1 and TGF-β3 levels were significantly reduced in AnxA1^−/−^ animals compared with their AnxA1⁺/⁺ counterparts (Supplementary Fig. S2).

### AnxA1 Modulates MAPKs and STAT3 Signaling Pathways

Given that AnxA1 deficiency selectively enhanced inflammatory mediators and markedly increased angiogenic and growth factors in the retina in DR, we next investigated whether these changes were associated with alterations in intracellular signaling pathways classically involved in inflammation, stress responses, and vascular dysfunction. To this end, the activation status of MAPK pathways and STAT3 signaling was evaluated in retinal homogenates.

**Figure 8.**
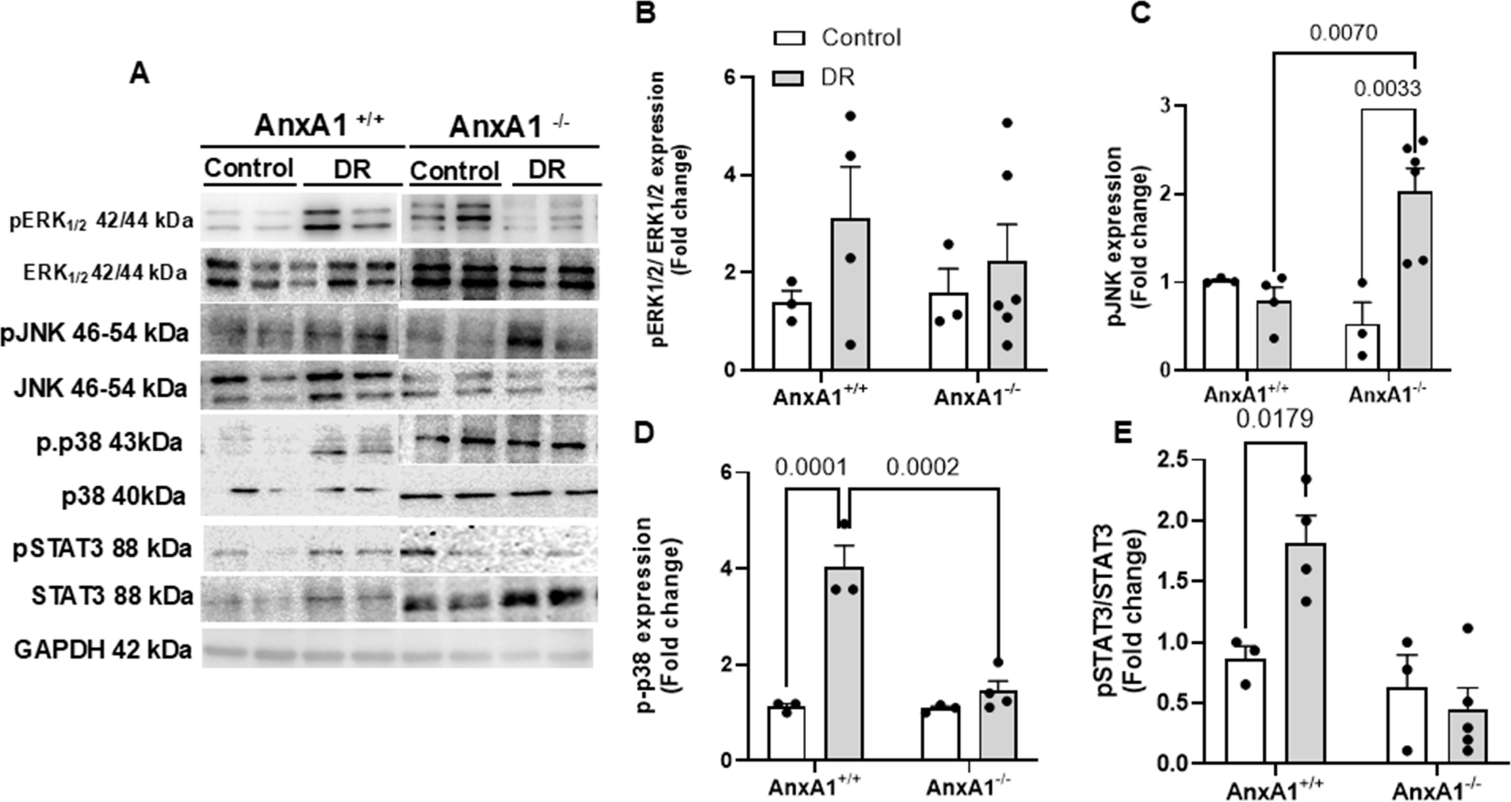
**The absence of AnxA1 deregulates the MAPK and STAT3 signaling pathways in DR.** (**A**) Representative immunoblots of protein expression of total and phosphorylated ERK (pERK), total and phosphorylated JNK (pJNK), total and phosphorylated p38 (p–p38), total and phosphorylated STAT3 (pSTAT3), and GAPDH-endogenous protein. (**B**–**E**) Data represent the mean ± SEM of phosphorylated/total protein expression (fold change), with dots representing individual animals (*n* = 3–5 animals/group, depending on sample availability). Statistical analysis was carried out using two-way ANOVA followed by Tukey's post hoc test.

To investigate the influence of AnxA1 on MAPK signaling pathways in DR, the expression of ERK, JNK, and p38 kinases was evaluated in the retinas (Figs. 8A–D). Analysis of ERK did not show its activation in the retinas between the different experimental conditions (Figs. 8A, 8B). However, deregulation of the JNK pathway was detected (Figs. 8A, 8B), indicated by the increased expression of pJNK in the retinas of AnxA1^−/−^ animals with DR compared with their controls (**P < 0.01) and compared with DR-induced AnxA1^+/+^ mice (**P < 0.01) (Figs. 7A, 7C). Activation of the p38 MAPK pathway was detected exclusively in AnxA1^+/+^ animals subjected to DR, with a significant increase in phosphorylated p38 levels compared with control animals (***P < 0.001) and with AnxA1^−/−^ animals with DR (***P < 0.001) (Figs. 8A, 8D).

The activation of the transcription factor STAT3, a signaling molecule associated with glial reactivity and GFAP regulation,^48^ was also investigated. AnxA1^+/+^ retinas in the DR group showed a significant increase in phosphorylated STAT3 levels compared with controls (**P < 0.01); no change was observed in AnxA1^−/−^ retinas (Figs. 8A, 8E).

## Discussion

This study investigated the role of endogenous AnxA1 in key processes associated with DR, including inflammation, Müller cell gliosis, angiogenic signaling, and retinal degeneration. Using the STZ-induced DR model together with a complementary CNV model, we demonstrated that the absence of AnxA1 is associated with the selective exacerbation of inflammatory and angiogenic mediators, dysregulation of MAPK and STAT3 signaling pathways, and increased retinal degeneration. In parallel, AnxA1 and its receptor FPR2 were upregulated in diabetic retinas, suggesting that the endogenous AnxA1/FPR2 axis participates in retinal responses during DR progression.

The metabolic characterization of the experimental model demonstrated sustained hyperglycemia and body weight loss in diabetic animals regardless of genotype. These findings are consistent with previous reports showing that AnxA1 deficiency does not markedly alter the establishment of hyperglycemia in experimental diabetes models.^49^^–^^51^ Indeed, studies using insulin resistance or high-fat diet–induced diabetes models have shown that AnxA1^−/−^ animals may exhibit impaired glucose tolerance without major differences in glycemic establishment compared with wild-type animals.^49^^–^^51^,^54^ Therefore, the molecular and retinal alterations observed in the present study are unlikely to result from differences in the systemic glycemic burden between strains.

DR is currently recognized as a chronic low-grade inflammatory disease in which metabolic stress, oxidative imbalance, and vascular dysfunction progressively induce inflammatory activation within the retina.^52^^,^^53^ In this context, our results demonstrated increased circulating T cells in both diabetic strains, whereas only AnxA1-deficient animals exhibited elevated systemic levels of IL-6, IL-17, CXCL1, and MCP-1. Previous studies in experimental ocular inflammation support an anti-inflammatory role for AnxA1. In allergic conjunctivitis, AnxA1 deficiency exacerbates leukocyte infiltration and cytokine production,^54^ while in autoimmune and endotoxin-induced uveitis models, the absence of AnxA1 intensifies inflammatory responses and increases IL-17 and cyclo-oxygenase-2 expression.^22^^,^^55^

Although classical inflammatory cytokines such as IL-1β and TNF-α were not broadly elevated in our experimental conditions, this does not exclude biologically relevant inflammatory dysfunction. Cytokine expression in STZ-induced DR models varies substantially according to disease stage, retinal compartment, and disease severity.^52^^,^^53^^,^^56^^,^^57^ In addition, DR inflammation is often characterized by temporally dynamic and selective cytokine modulation rather than generalized cytokine amplification. Therefore, our findings suggest that endogenous AnxA1 deficiency promotes selective inflammatory dysregulation rather than a broad inflammatory response, as evidenced by altered leukocyte dynamics, increased circulating IL-6, IL-17, CXCL1, and MCP-1, together with retinal gliosis and enhanced angiogenic signaling.

Besides systemic inflammation, retinal neurodegeneration is increasingly recognized as an early and critical component of DR pathogenesis. In the present study, AnxA1-deficient diabetic animals exhibited retinal thinning and reduced photoreceptor-associated gene expression, particularly involving rhodopsin. Previous studies from our group demonstrated that endogenous AnxA1 is essential for maintaining retinal electrophysiological homeostasis.^34^ Moreover, AnxA1 exerts neuroprotective effects in models of diffuse axonal injury by reducing oxidative stress, inflammatory cytokine production, and neuronal apoptosis.^58^ In microglia, AnxA1 deficiency impairs the recognition and clearance of apoptotic neurons, contributing to neuroinflammatory imbalance.^59^ Collectively, these findings support the notion that endogenous AnxA1 contributes to the preservation of retinal neurons during diabetes-associated stress.

Müller cell gliosis represents another hallmark of DR progression and is closely associated with neurovascular dysfunction and chronic retinal inflammation.^10^^,^^60^ In the present study, diabetic wild-type animals exhibited marked GFAP and vimentin upregulation, predominantly in Müller cell processes and endfeet, whereas AnxA1-deficient retinas showed attenuated gliosis despite enhanced inflammatory and angiogenic alterations. This apparent dissociation suggests that inflammatory activation and Müller cell gliosis may not occur in parallel under all experimental conditions.

Previous studies have shown that FPR2-deficient animals exhibit attenuated retinal gliosis,^13^ indicating that the AnxA1/FPR2 axis may directly participate in glial activation. In agreement with this concept, we observed increased retinal AnxA1 and FPR2 expression in diabetic wild-type animals, particularly in regions associated with Müller cell activation. The STAT3 signaling pathway is a major regulator of reactive gliosis and directly controls GFAP transcription.^61^ Interestingly, STAT3 activation was observed only in AnxA1^+/+^ diabetic retinas, whereas AnxA1-deficient animals failed to activate this pathway, which may explain the absence of robust GFAP induction in these animals. Together, these findings suggest that endogenous AnxA1 may differentially regulate inflammatory and glial responses during DR.

The in silico analyses further supported the relevance of AnxA1 in DR progression. We observed increased *Anxa1* expression in experimental DR and a positive association between *ANXA1* expression and disease severity in human retinal datasets. Furthermore, both GFAP and ANXA1 were preferentially elevated in the peripheral retina, a region more susceptible to ischemic stress during DR progression.^62^ Because Müller cells are among the main retinal sources of AnxA1,^21^ these findings reinforce the hypothesis that AnxA1 expression is associated with retinal stress responses and glial activation.

At the retinal level, AnxA1 deficiency selectively increased MCP-1 and IL-4 levels. MCP-1 is a major chemokine involved in leukocyte recruitment, vascular permeability, and retinal inflammation during DR.^63^ Increased MCP-1 expression has been associated with the breakdown of the blood–retinal barrier and VEGF induction under hyperglycemic conditions. Accordingly, the elevated MCP-1 levels observed in AnxA1-deficient retinas were accompanied by increased VEGF-A, endothelin-1, and EGF levels, supporting a selective proangiogenic signaling profile independent of gliosis activation.

Our findings demonstrate that, in the retinas of AnxA1^−/−^ animals, but not in wild-type animals with DR, there is a marked increase in the expression of IL-4 and MCP-1, potentially secreted by Müller cells. The significant elevation in IL-4 levels in AnxA1^−/−^ animals suggests the activation of specific immune pathways linked to the production of this cytokine. IL-4 is a key modulator of the immune response, particularly in promoting a T helper cell 2–type immune response.^64^ In the retina, IL-4 exerts antiproliferative effects, promotes the differentiation of photoreceptors and glial cells, supports axonal regeneration, and possesses neuroprotective and anti-inflammatory properties.^65^^–^^68^ Furthermore, IL-4 has been detected in the vitreous humor of patients with DR.^69^ These findings offer valuable insights into the immunological mechanisms underlying DR, suggesting that the absence of AnxA1 in this disease context stimulates IL-4 production, which may play a protective role against inflammation-driven retinal damage.

MCP-1 plays a crucial role in the induction of fibrosis and angiogenesis by increasing VEGF production.^70^ In line with this, AnxA1-deficient retinas exhibited increased MCP-1 levels, accompanied by elevated expression of VEGF-A, endothelin-1, and EGF in the DR model. These findings support an association between endogenous AnxA1 deficiency and a proangiogenic signaling profile during DR progression. Although VEGF-A was selectively increased in AnxA1^−/−^ diabetic animals, no significant alterations were detected in VEGF-C, VEGF-D, or FGF-2 levels in the DR model, suggesting that AnxA1 deficiency does not globally amplify all angiogenic mediators under these experimental conditions.

Previous studies demonstrated that VEGF signaling may depend on the AnxA1/FPR pathway.^71^ In addition, the Ac_2–26_ peptide derived from AnxA1 has been shown to modulate angiogenic responses in other tissues.^72^ Because VEGF remains the primary therapeutic target for proliferative DR and retinal neovascular diseases,^73^^,^^74^ our findings reinforce the relevance of the endogenous AnxA1/FPR2 axis in pathways associated with retinal vascular dysfunction.

The CNV model was used as a complementary angiogenic paradigm to further investigate the contribution of endogenous AnxA1 to angiogenesis-associated signaling. AnxA1-deficient animals exhibited selective upregulation of VEGF-C, whereas no significant alterations were observed in VEGF-A, VEGF-D, endothelin-1, EGF, or FGF-2 levels. Previous studies demonstrated an overexpression of the AnxA1/FPR2 axis in the choroid during CNV formation,^75^ supporting the involvement of this pathway in neovascular responses. Because CNV involves laser-induced tissue injury and wound-healing responses, these findings should be interpreted as complementary evidence of AnxA1-mediated angiogenic signaling rather than as direct evidence of the mechanisms underlying retinal neovascularization in DR.

Endothelin-1 emerged as another relevant mediator selectively altered in the DR model. This vasoactive peptide is strongly implicated in retinal vascular dysfunction and is elevated in different stages of DR, including nonproliferative DR and proliferative DR.^76^^–^^80^ Moreover, endothelin-1 may directly regulate AnxA1 stability through carbonylation-dependent proteasomal degradation.^81^ Therefore, the increased endothelin-1 levels observed specifically in AnxA1-deficient diabetic animals may contribute to the vascular dysregulation associated with retinal inflammation and angiogenic stress.

Regarding TGF-β signaling, no significant alterations were detected among the three TGF-β isoforms in the DR model. In contrast, in the CNV model, AnxA1 deficiency was associated with reduced levels of TGF-β1 and TGF-β3, whereas TGF-β2 levels remained unchanged. Because TGF-β isoforms exert distinct effects on endothelial homeostasis, oxidative stress responses, and angiogenesis-associated signaling,^82^ these findings suggest that endogenous AnxA1 may contribute to the regulation of selective TGF-β pathways during pathological neovascularization rather than during early-to-intermediate stages of DR.

MAPK signaling pathways are critically involved in cellular responses to hyperglycemia, oxidative stress, inflammation, and angiogenesis.^11^^,^^83^ In the present study, ERK activation was not significantly altered under the evaluated conditions. In contrast, JNK activation was selectively increased in AnxA1-deficient diabetic retinas, whereas p38 activation was observed exclusively in wild-type diabetic animals. Previous studies have demonstrated that JNK and p38 signaling contribute to retinal degeneration, oxidative stress, inflammatory responses, and angiogenesis during DR progression.^84^^,^^85^ Therefore, our findings suggest that endogenous AnxA1 differentially modulates MAPK pathway activation during diabetic retinal stress, particularly involving JNK- and p38-associated responses.

Some considerations regarding the scope of the present study are worth noting. Although our data identify important molecular and signaling alterations associated with endogenous AnxA1 deficiency, we acknowledge that functional assessments (e.g., retinal vascular leakage, ERG, visual function analysis, and quantitative CNV measurements) were not included, and the inflammatory changes observed were selective rather than reflecting a broad upregulation of canonical cytokines. Therefore, our findings should be interpreted primarily as evidence of selective alterations in proangiogenic signaling in the STZ-induced DR model. In addition, because CNV does not reproduce proliferative diabetic retinal neovascularization, future studies using retinal neovascularization models will be necessary to establish the direct translational relevance of AnxA1-mediated angiogenic signaling to advanced DR.

## Conclusions

Our findings show that the absence of AnxA1 leads to the increased expression of proangiogenic factors and specific cytokines in the diabetic retina. Conversely, AnxA1 deficiency is also associated with reduced retinal gliosis, suggesting a dual, apparently paradoxical effect. This body of evidence positions AnxA1 as a central modulator of inflammation, proangiogenic signaling, and glial responses in DR, highlighting the complexity of its pathophysiological role and its potential as a therapeutic target that warrants careful investigation.

## Supplementary Material

Supplement 1

Supplement 2
