# Supplementary material for: Annexin A1 as a Key Modulator of Inflammatory, Glial, and Angiogenic Signaling Pathways in Diabetic Retinopathy
Source: Invest Ophthalmol Vis Sci. 2026 Aug 5;67(10):12. doi: 10.1167/iovs.67.10.12 (PMC13450646; doi:10.1167/iovs.67.10.12)

Figure 4 I and L

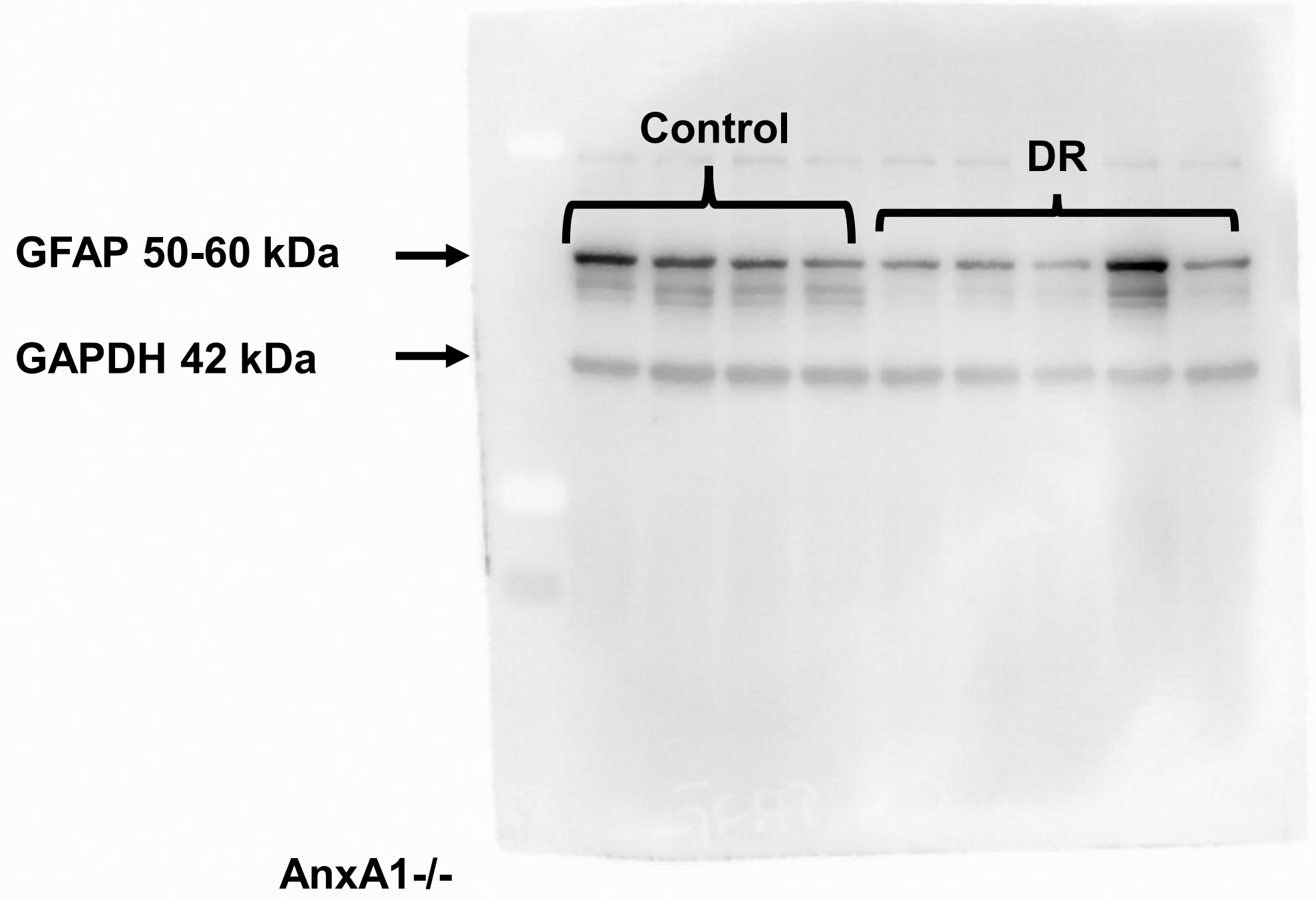

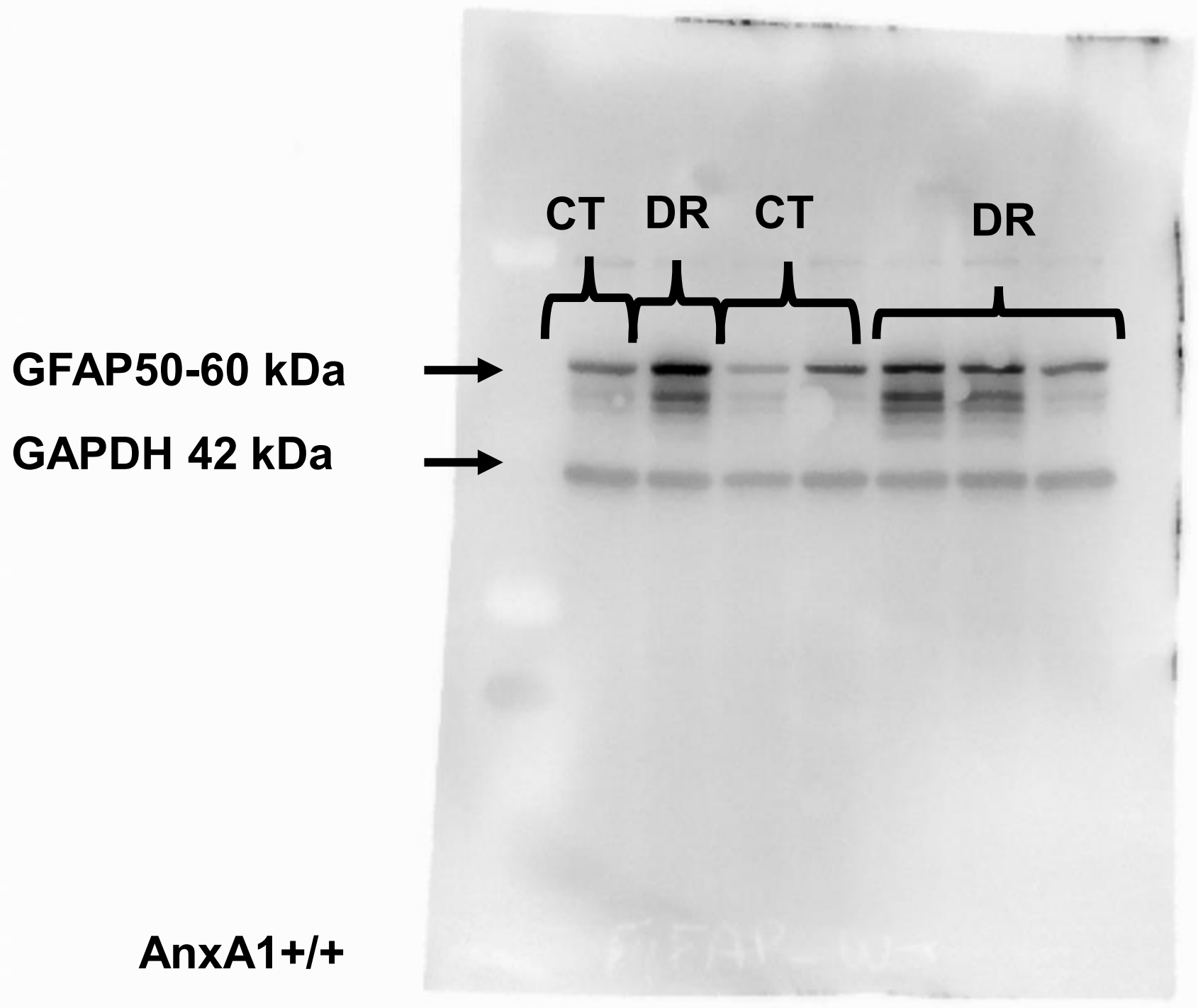

Figure 4 I and M

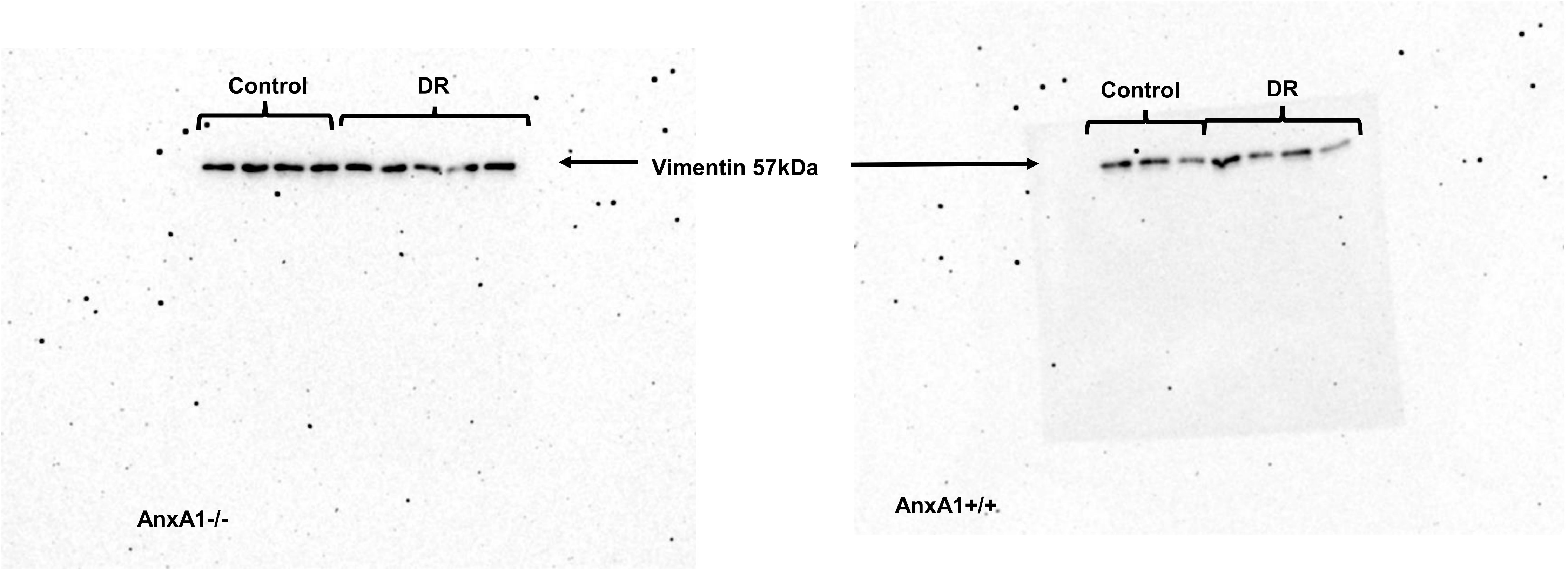

Figure 4 I and N

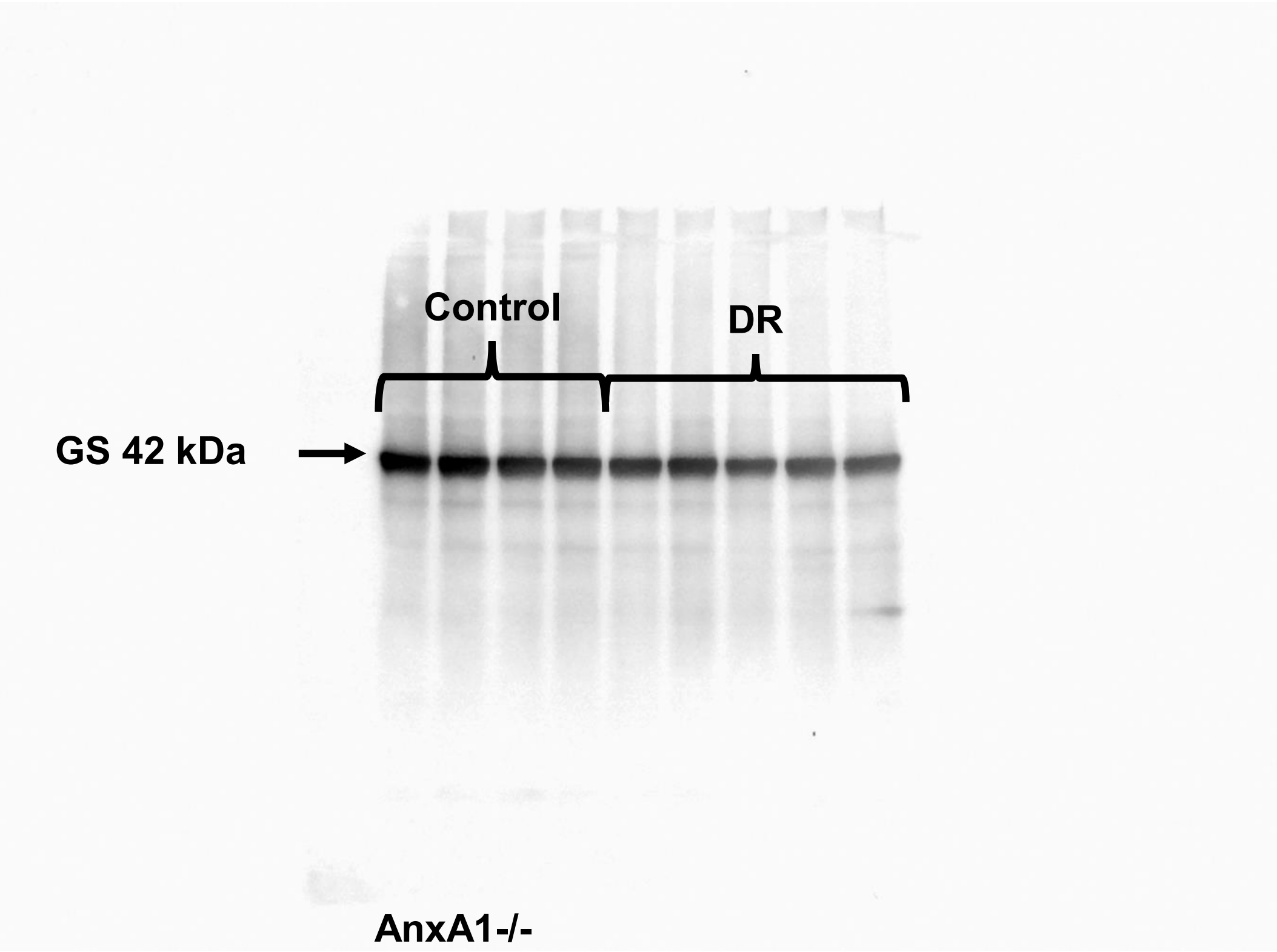

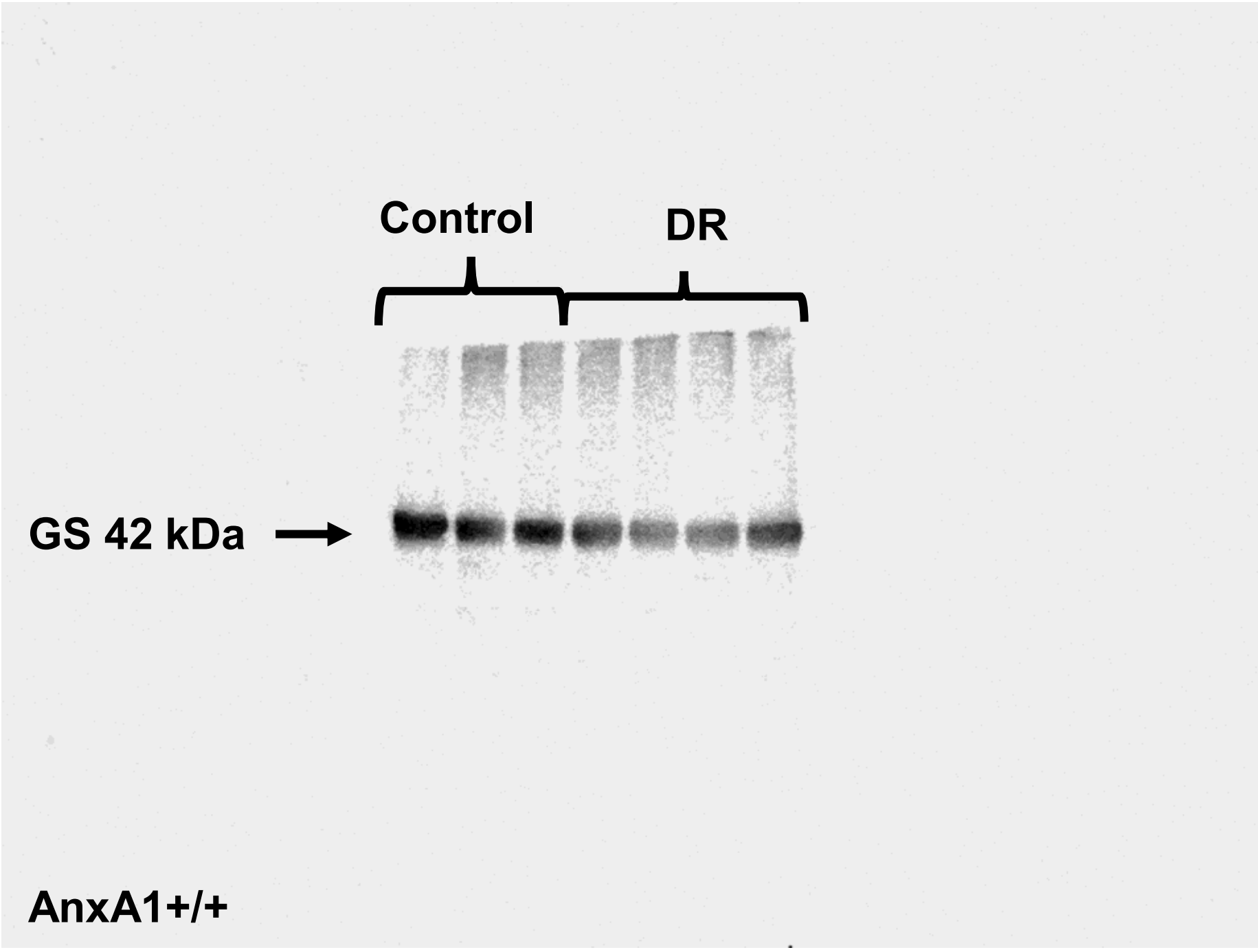

Figure 5 A and B

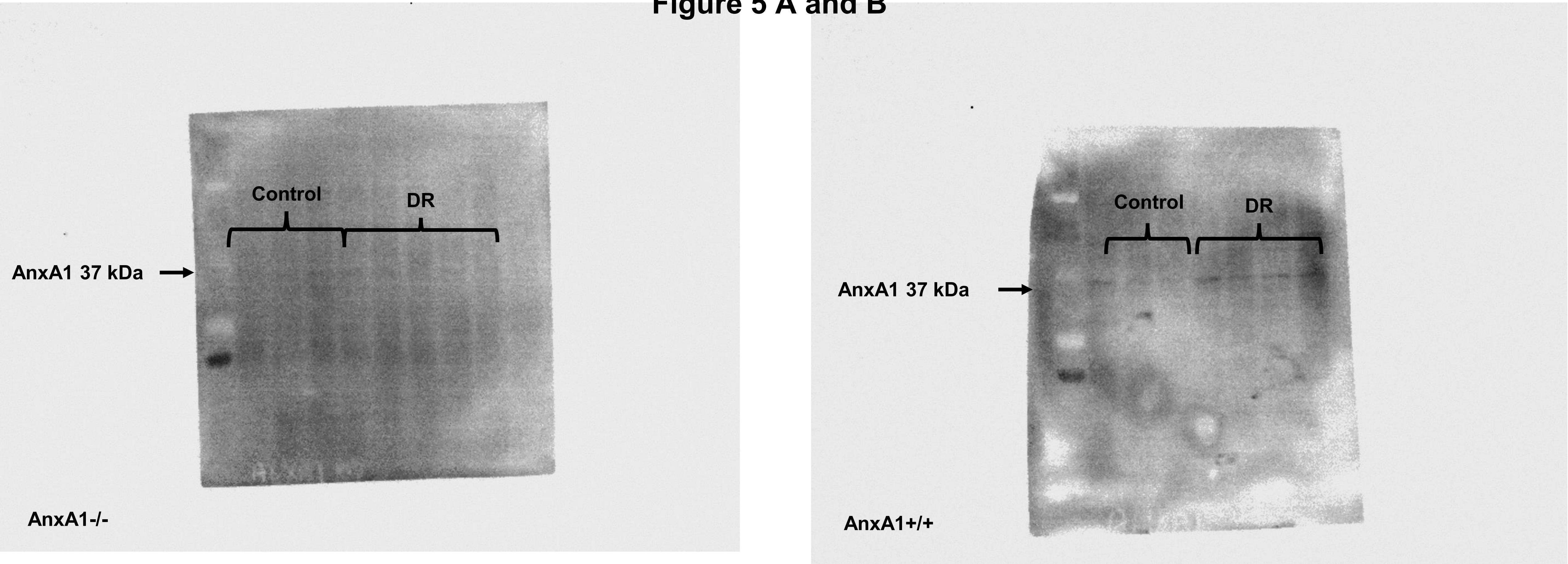

Figure 5 A and C

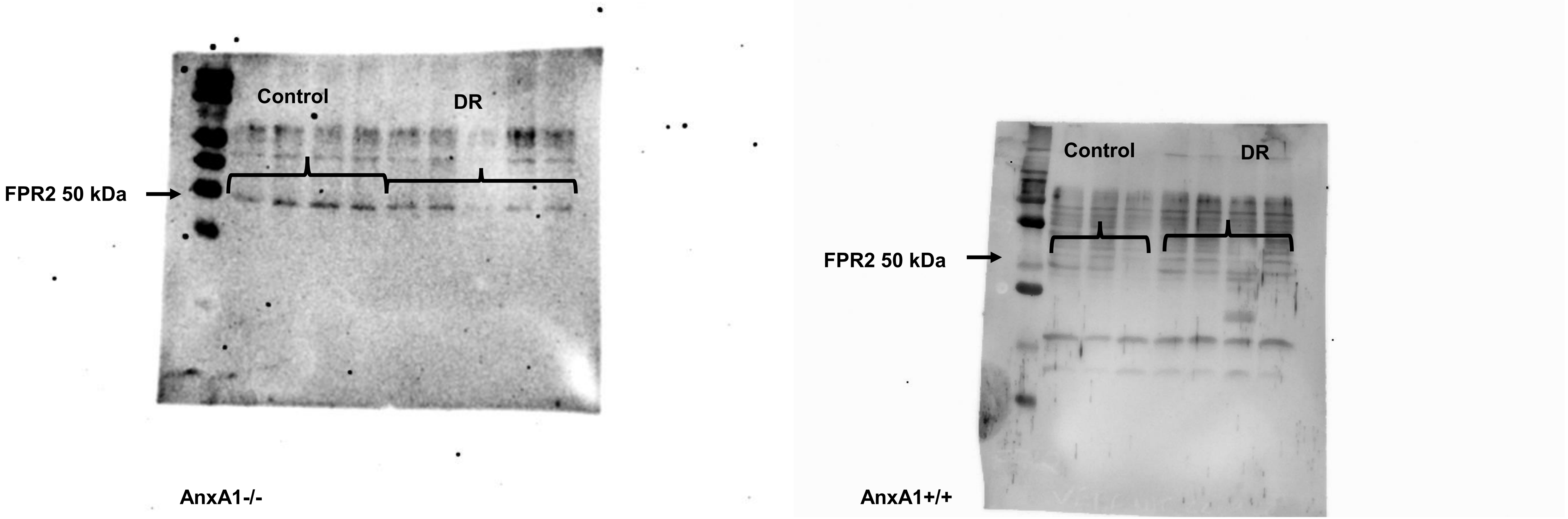

Figure 8 A and B

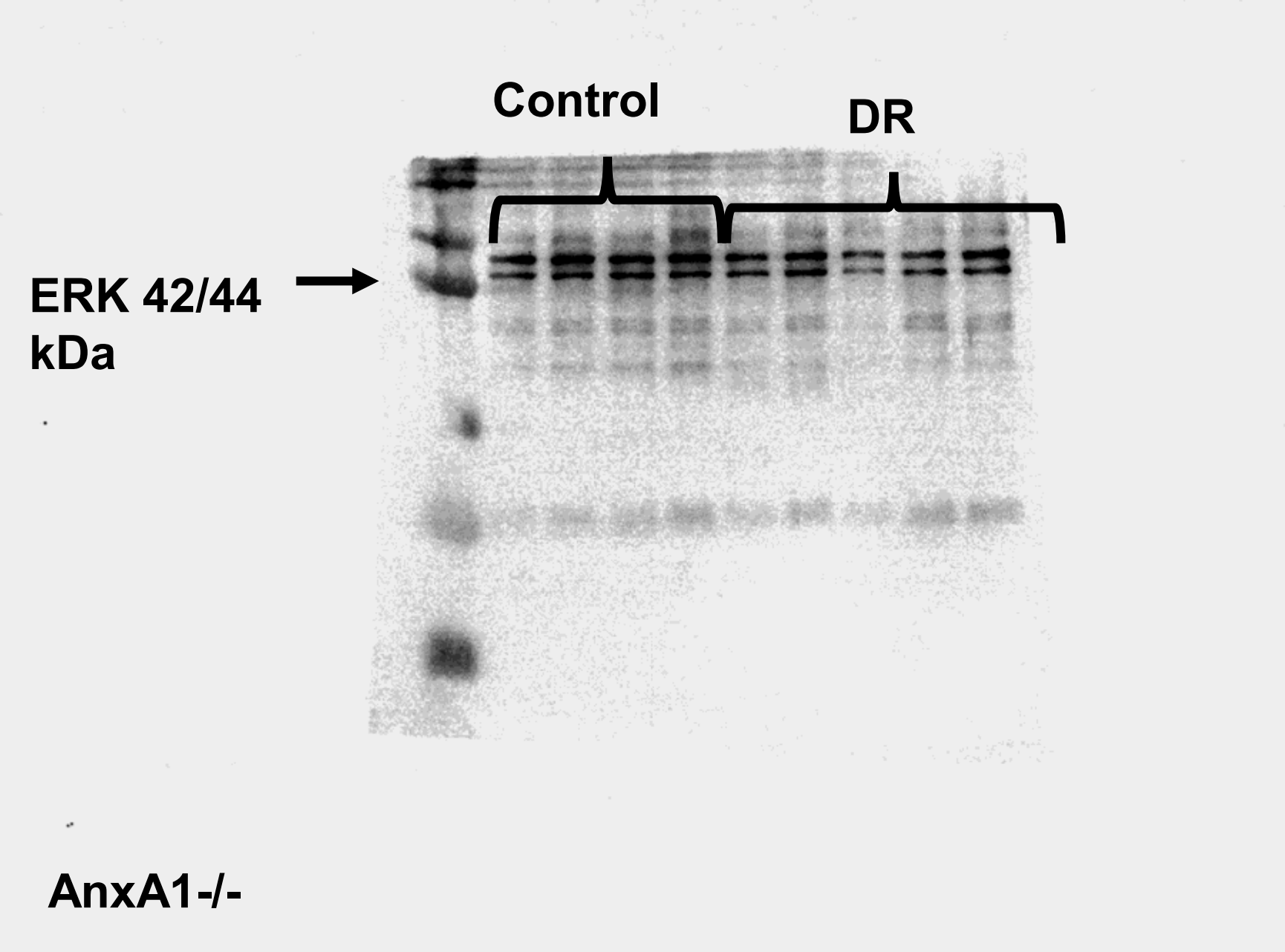

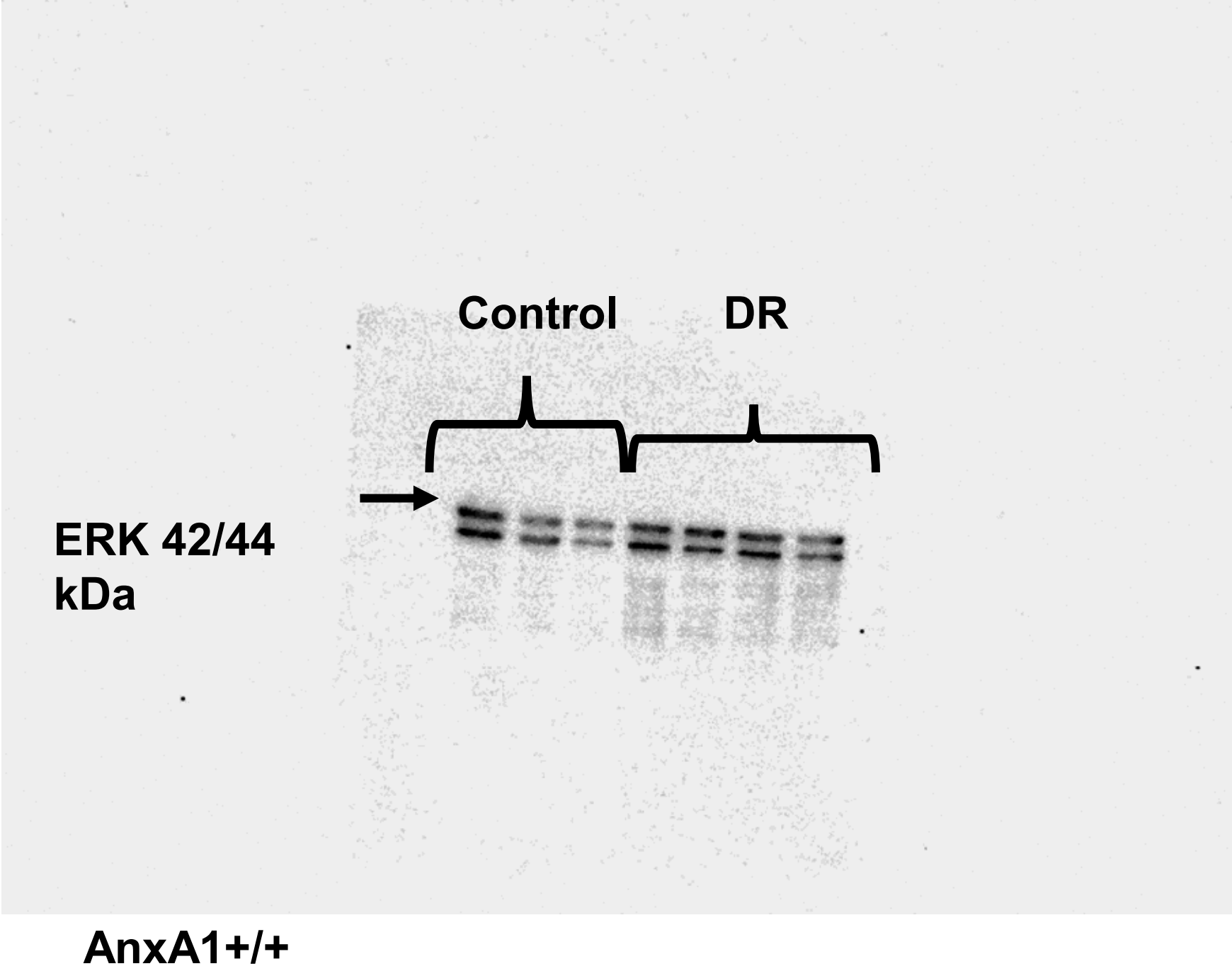

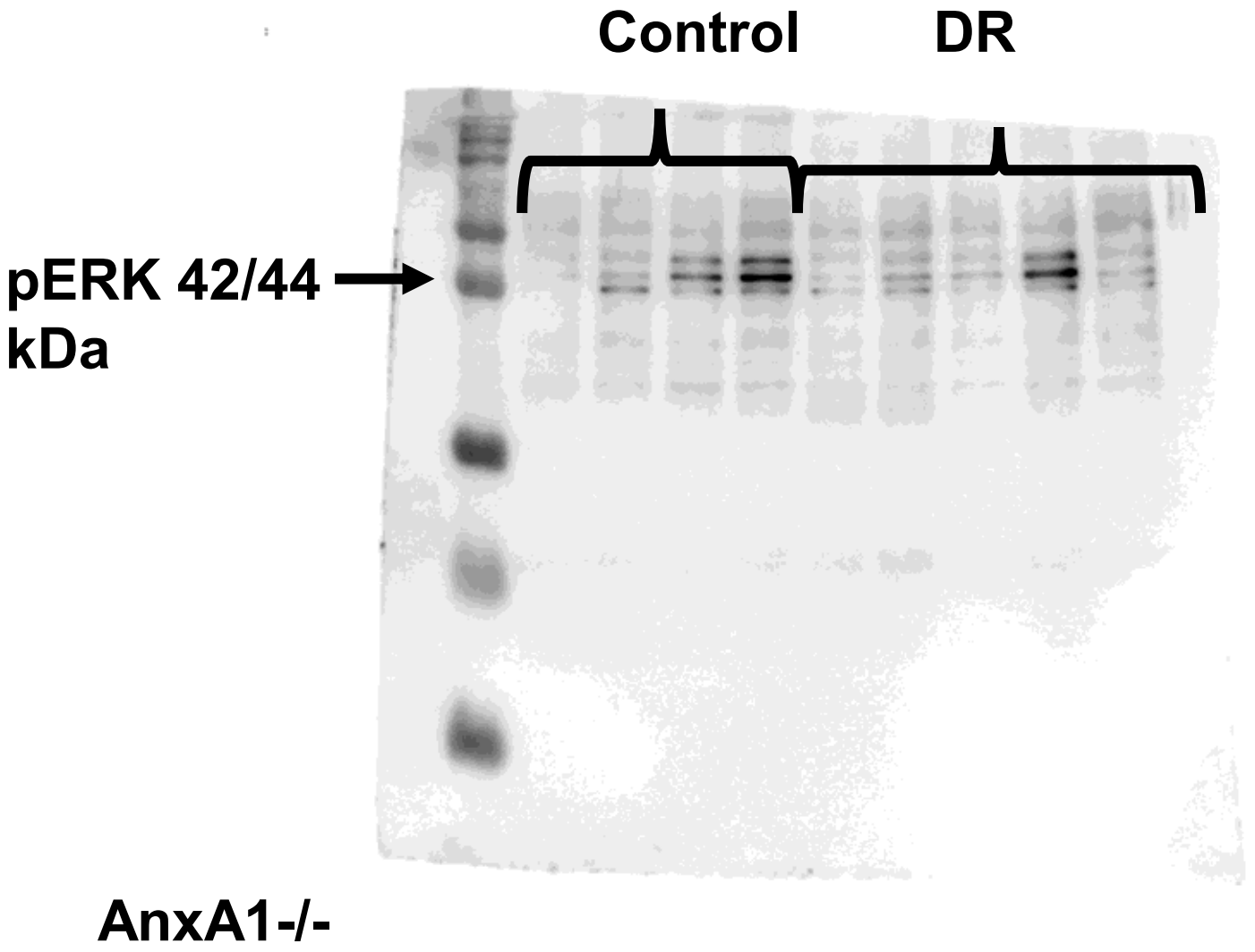

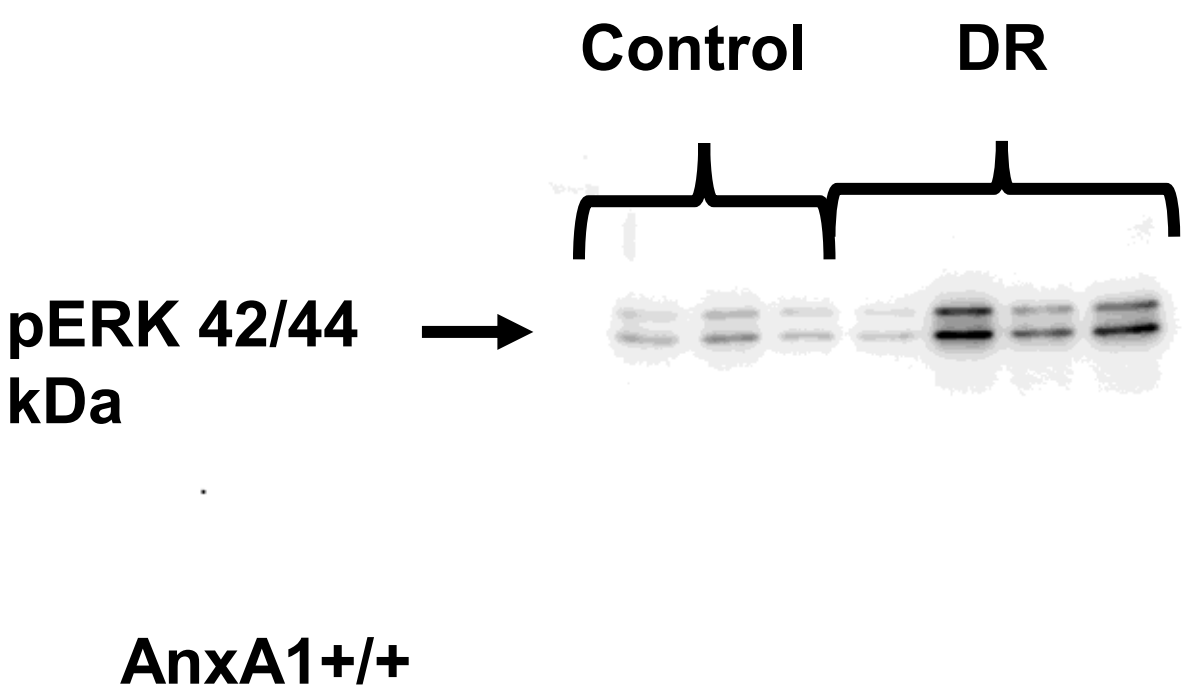

Figure 8 A and C

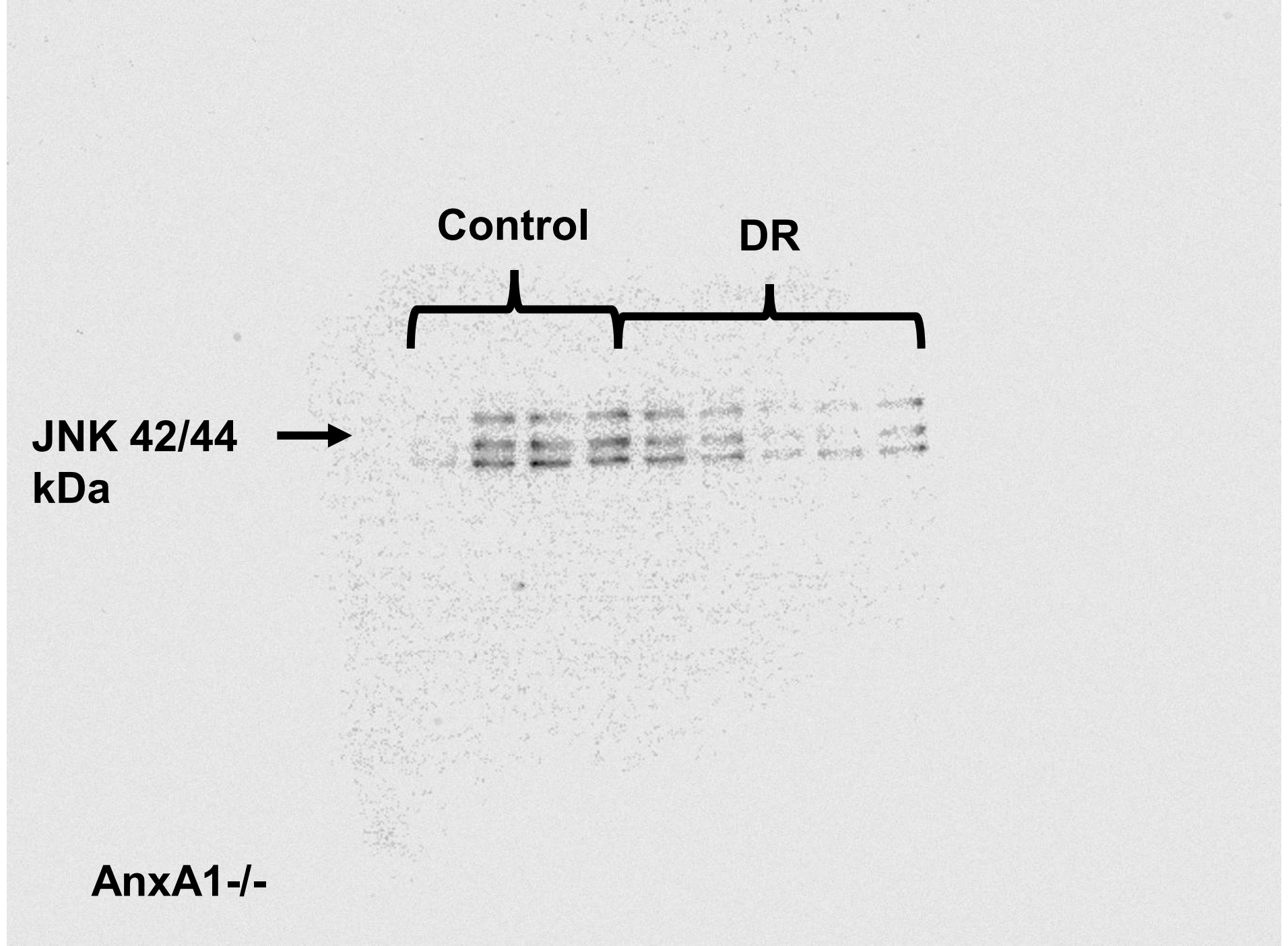

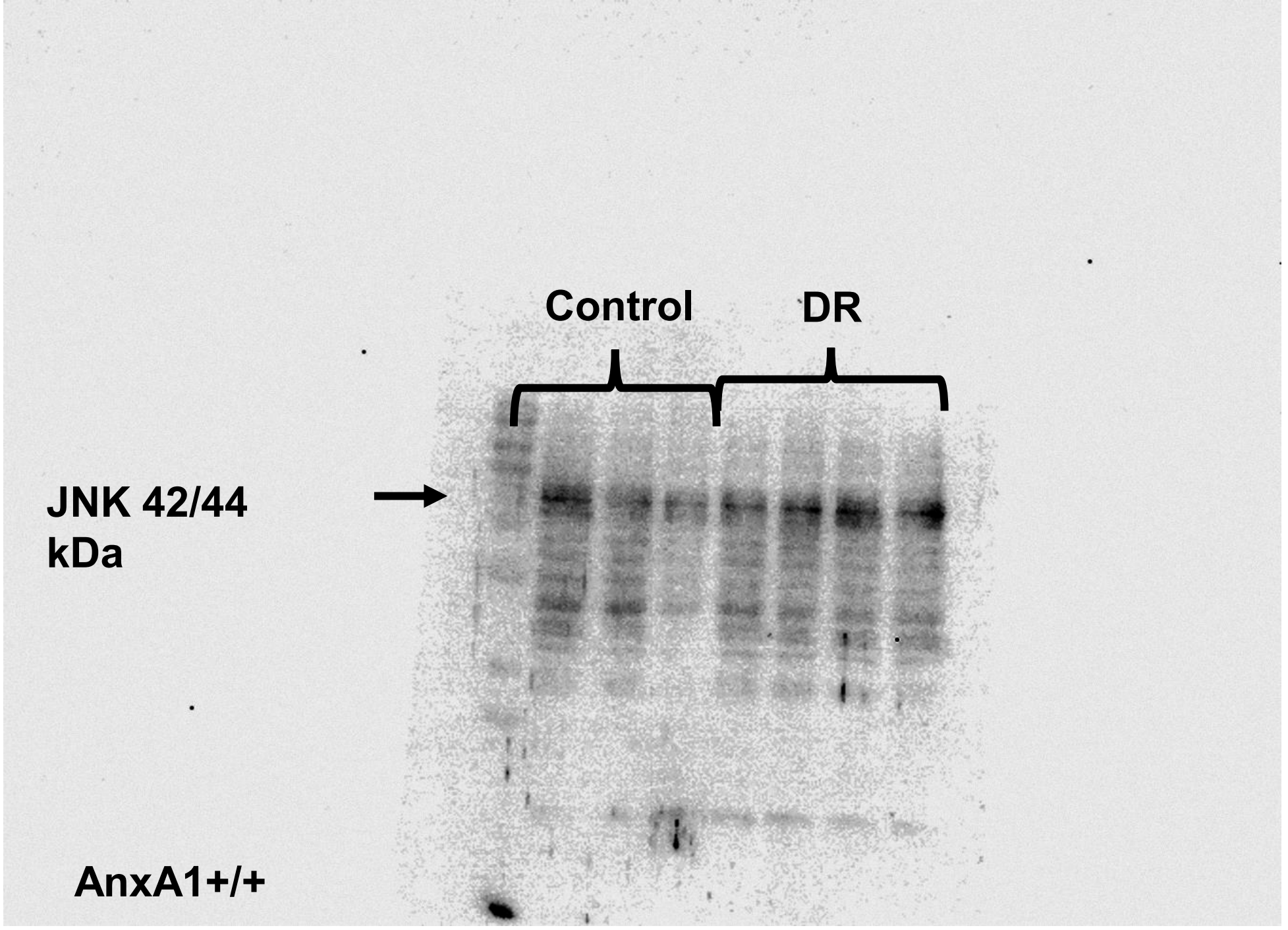

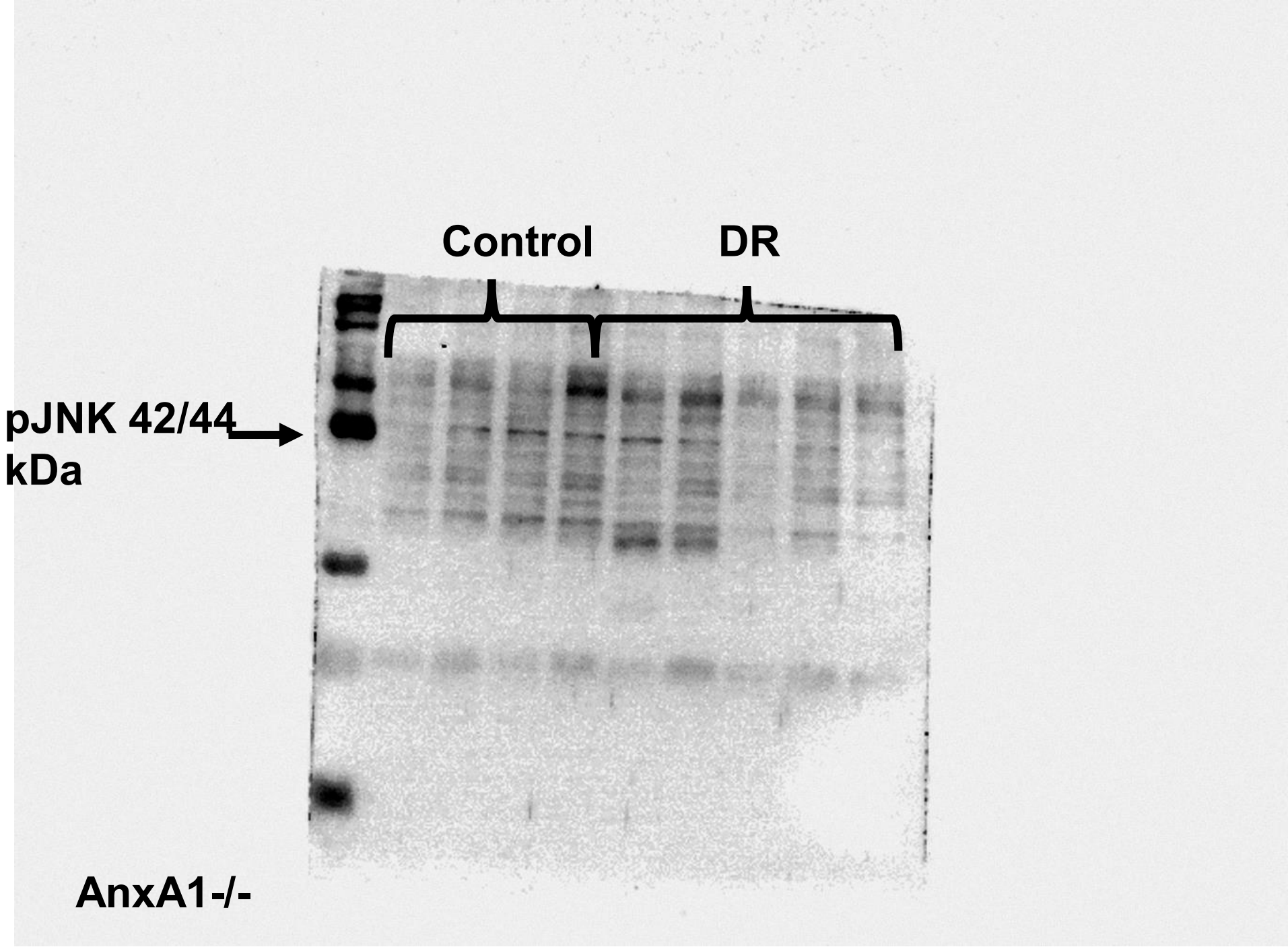

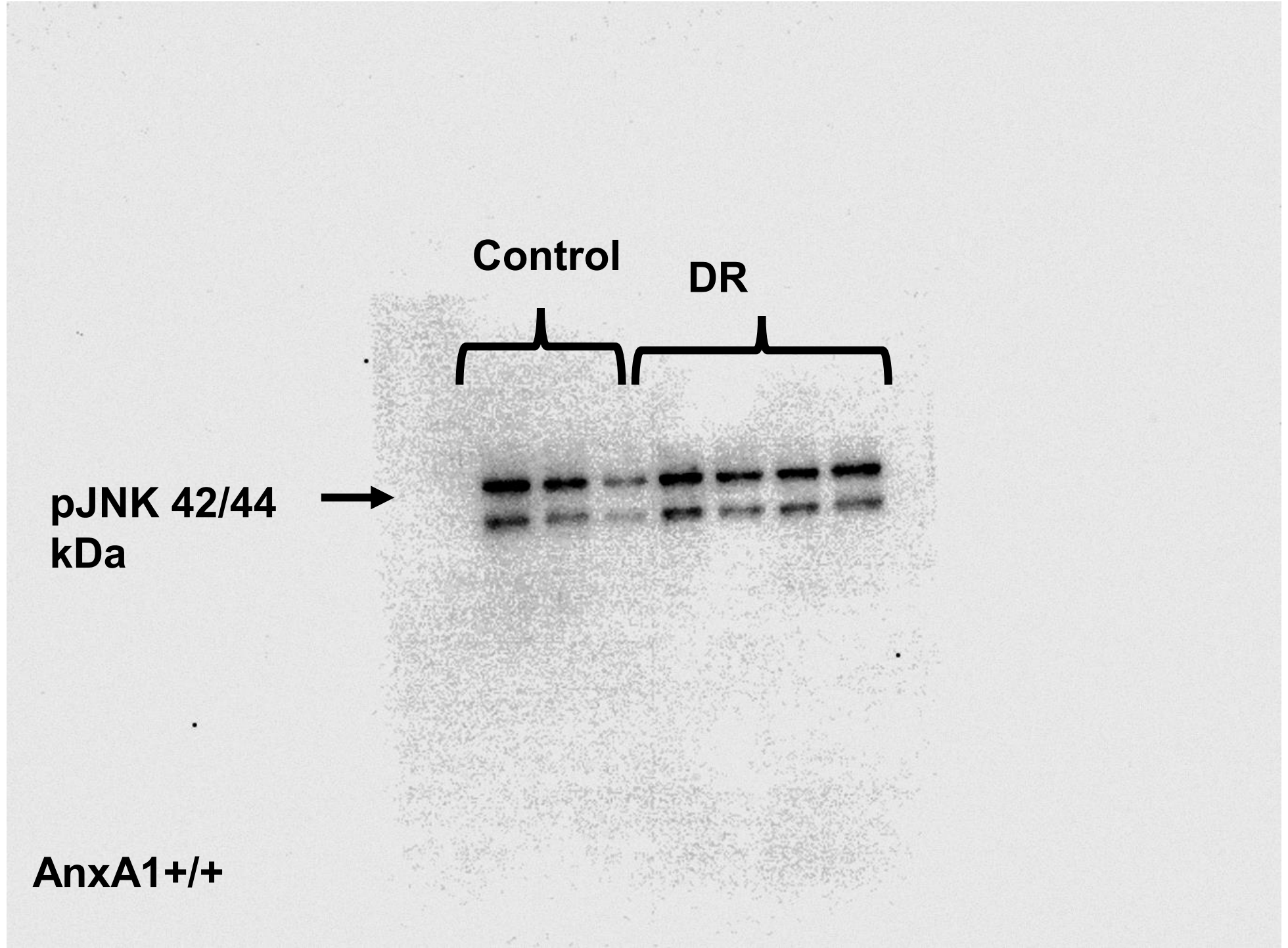

Figure 8 A and D

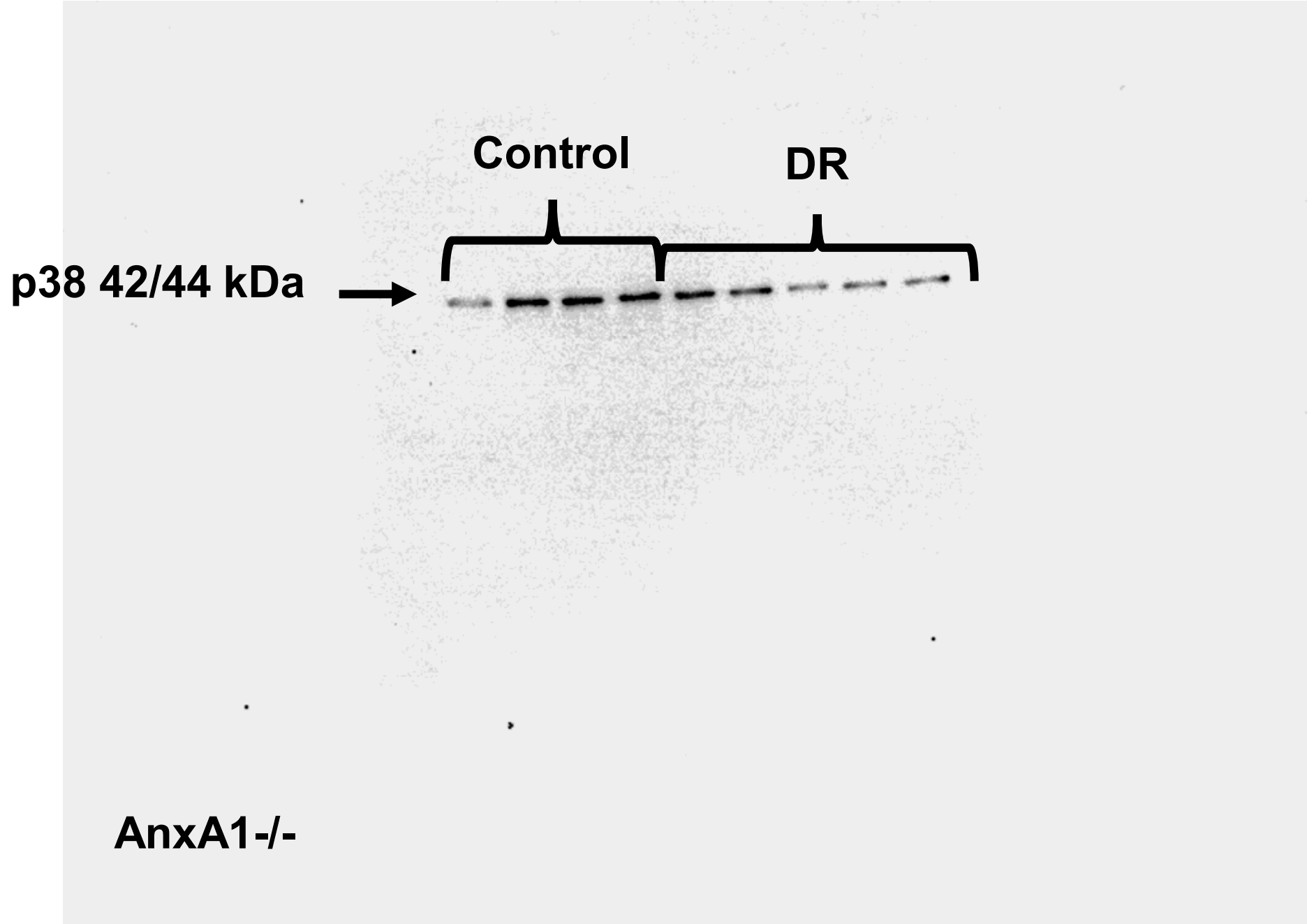

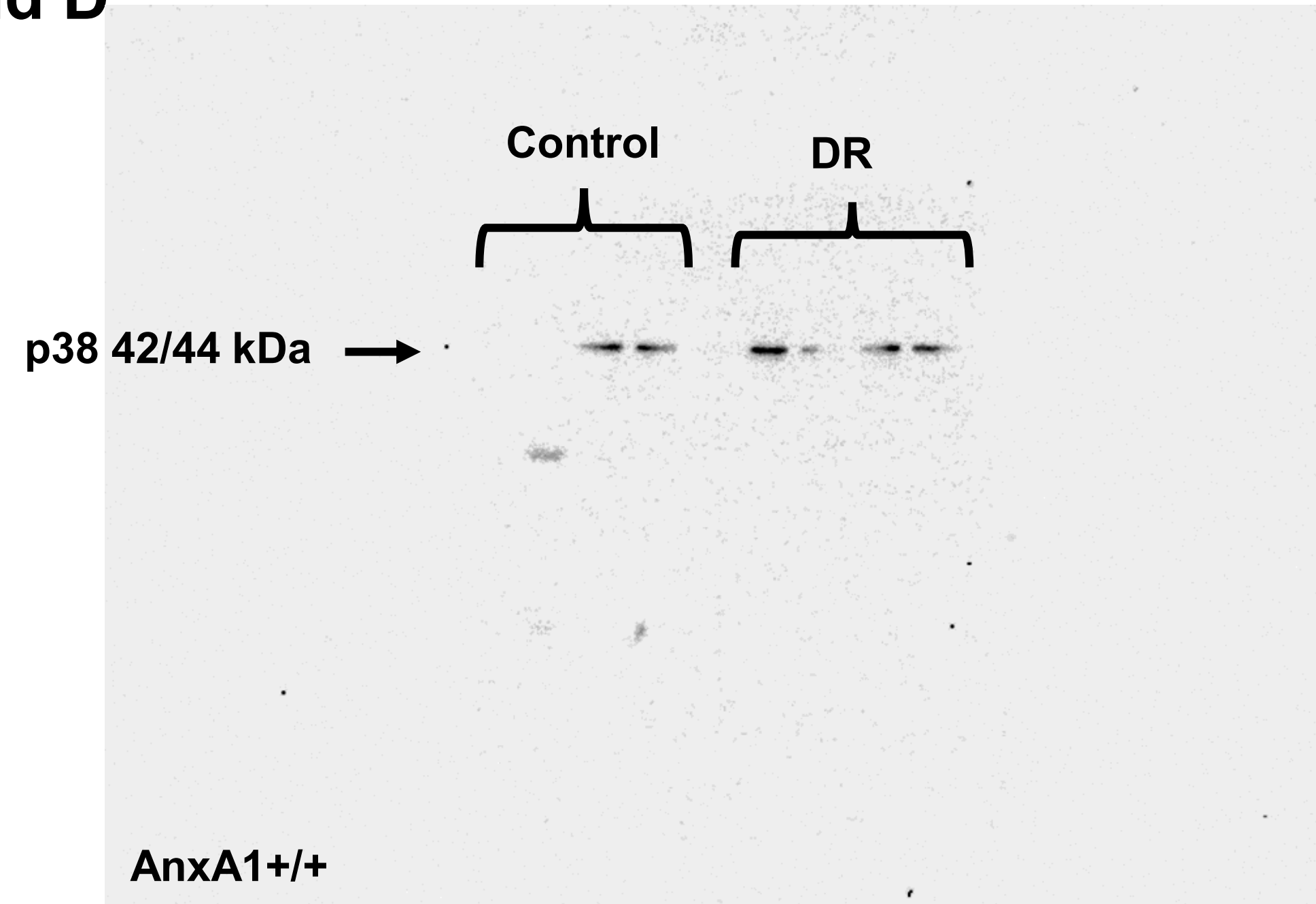

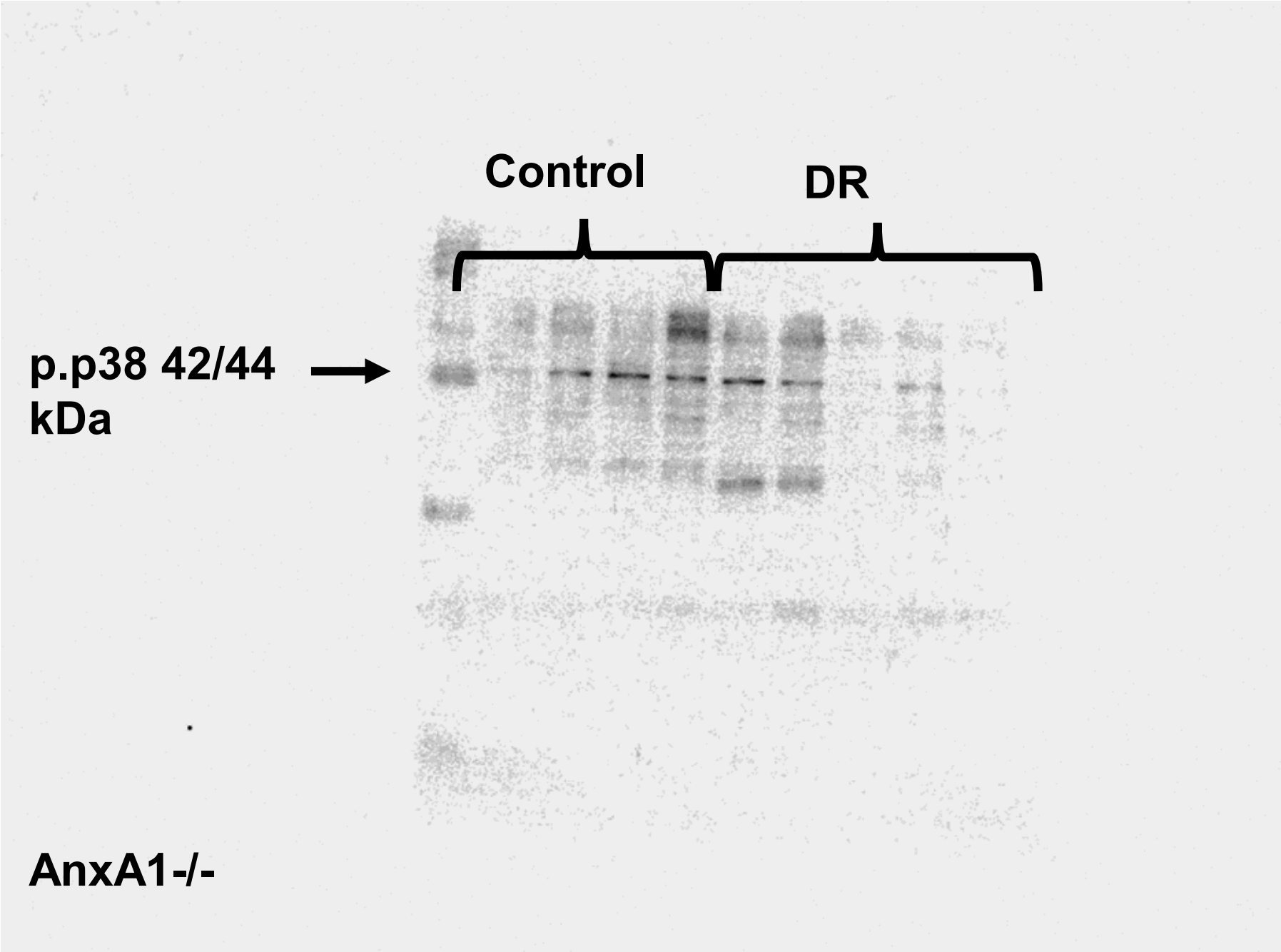

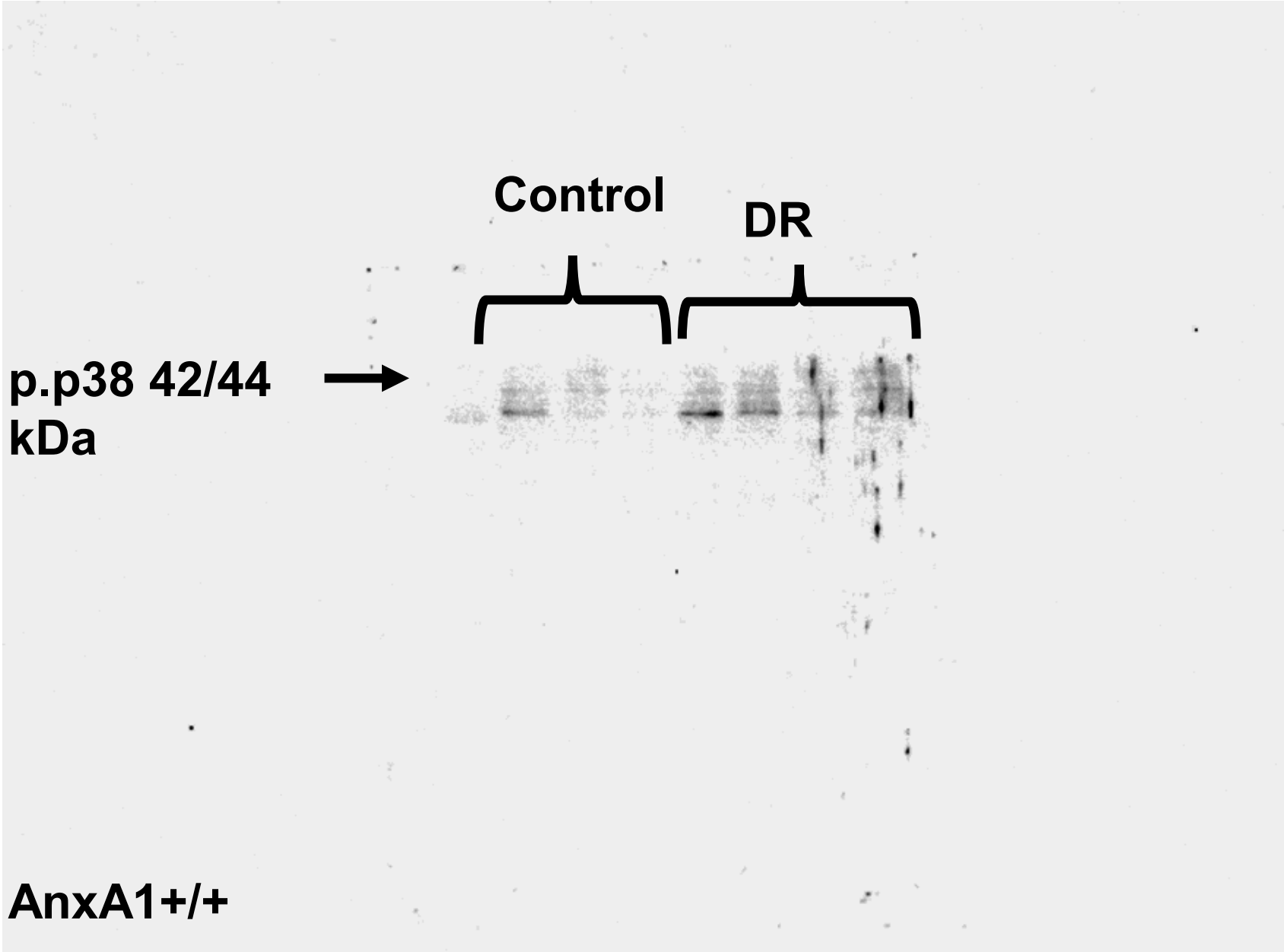

Figure 8 A and E

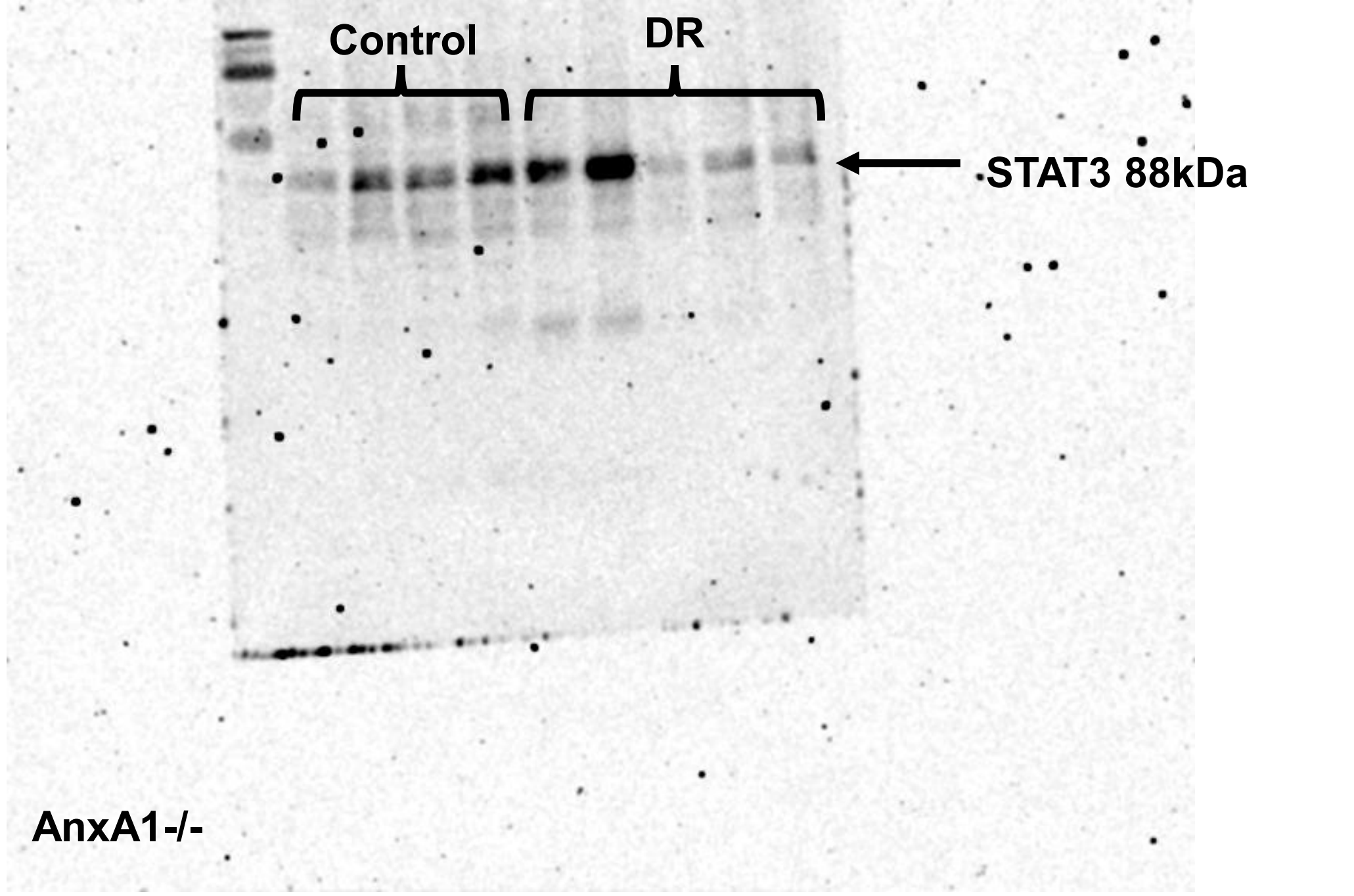

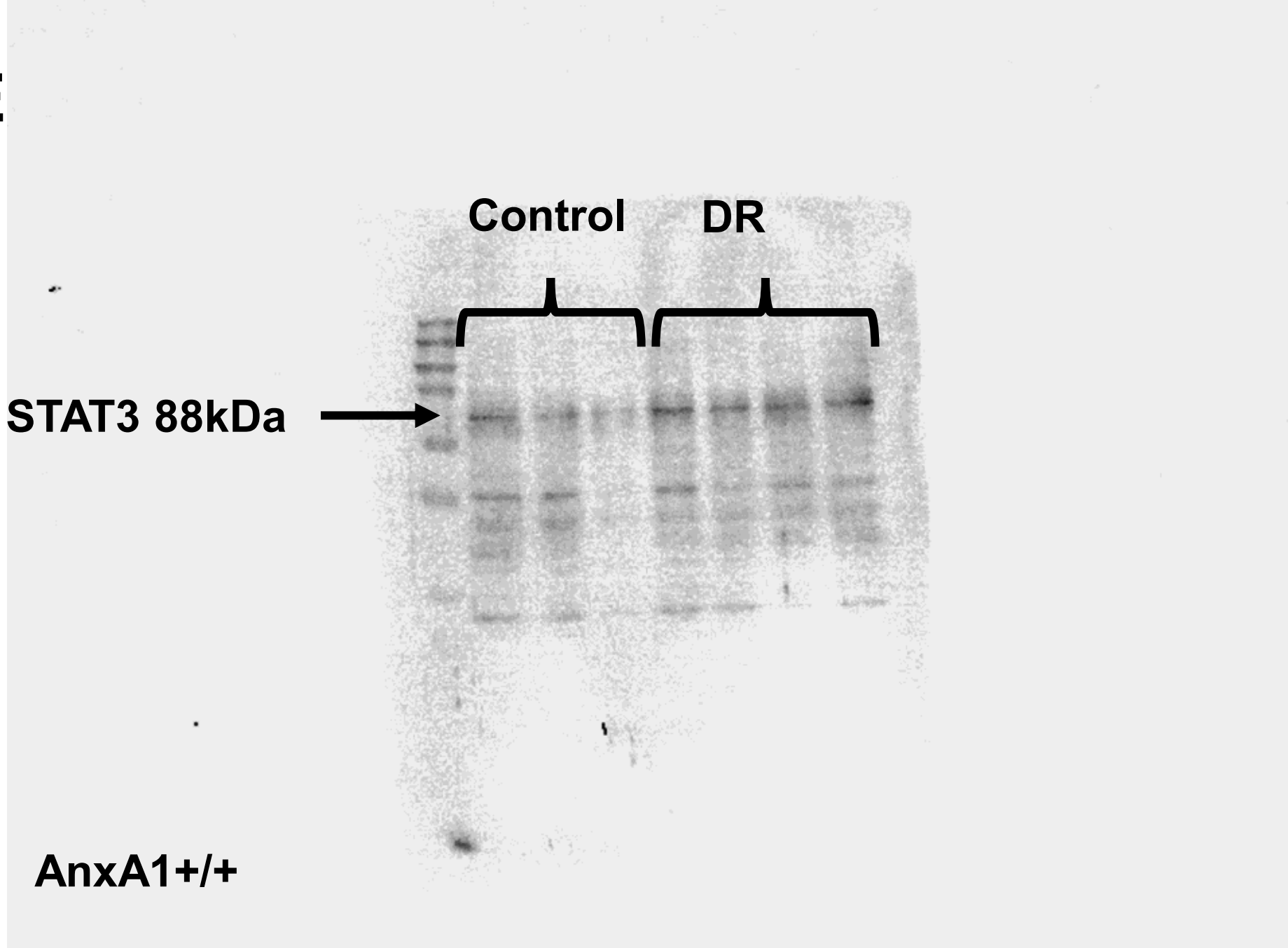

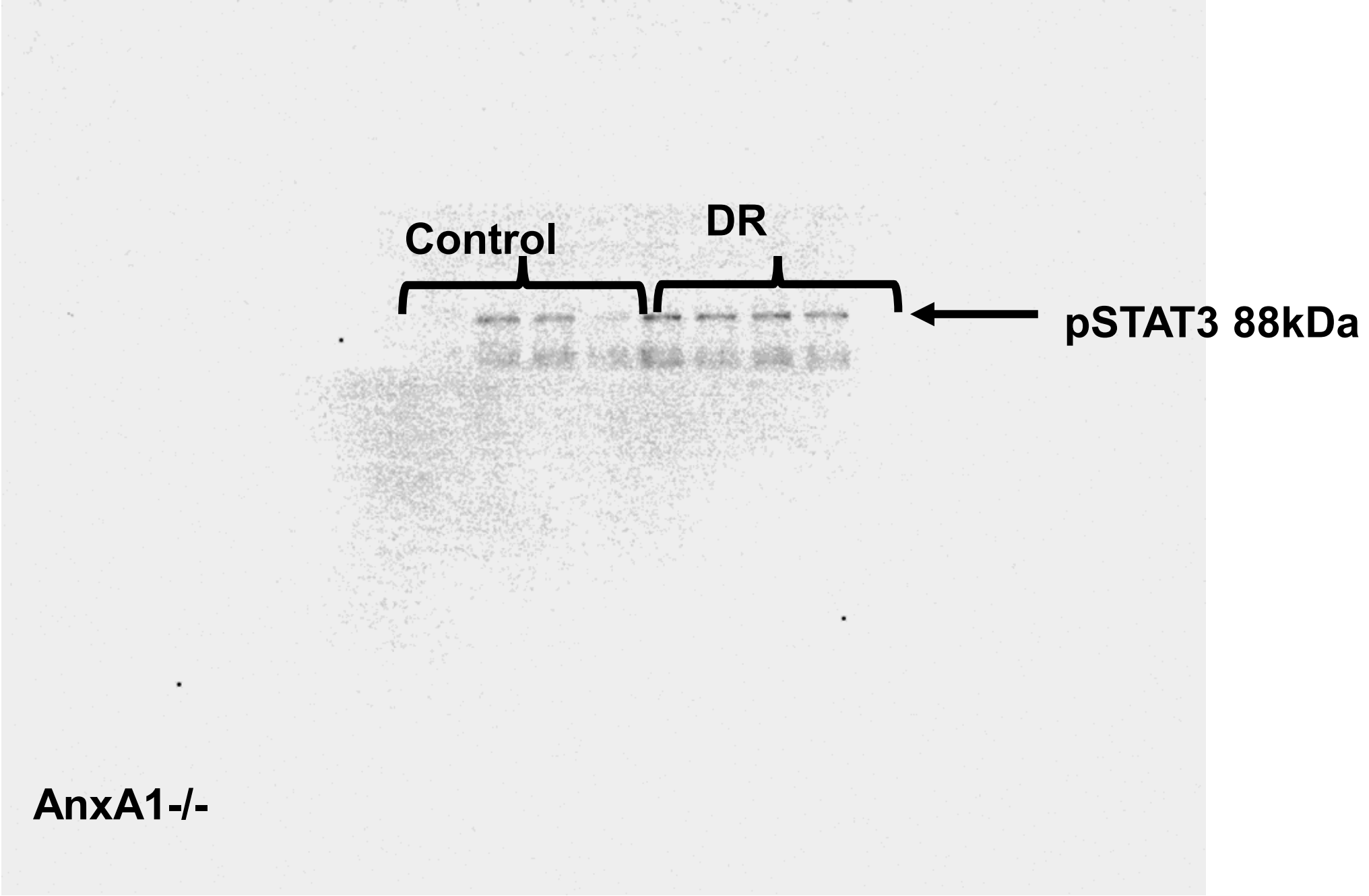

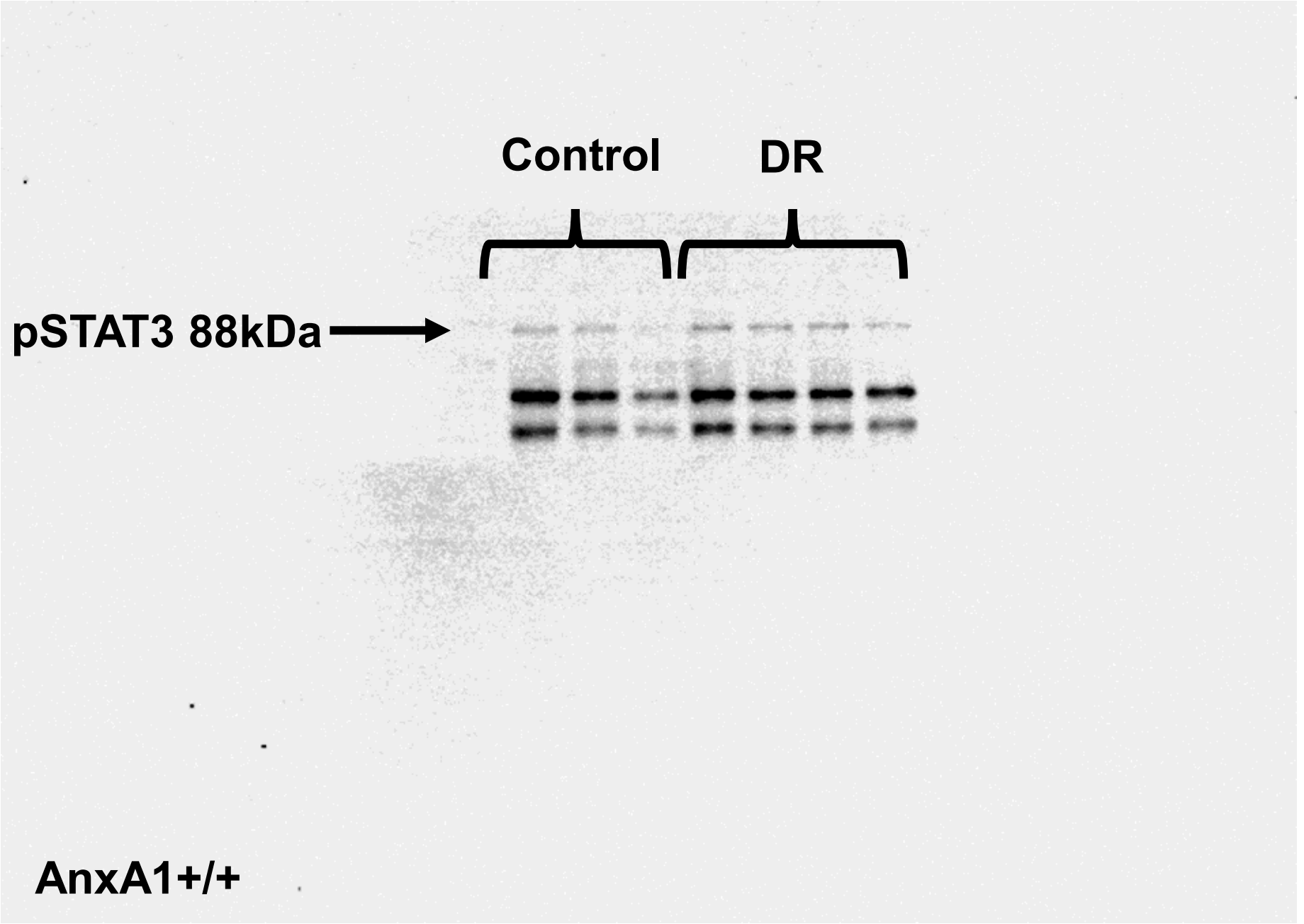

Supplement: Supplementary file 1 [file iovs-67-10-12_s002.pdf]
