# Supplementary material for: Annexin A1 as a Key Modulator of Inflammatory, Glial, and Angiogenic Signaling Pathways in Diabetic Retinopathy
Source: Invest Ophthalmol Vis Sci. 2026 Aug 5;67(10):12. doi: 10.1167/iovs.67.10.12 (PMC13450646; doi:10.1167/iovs.67.10.12)
Supplement: Supplementary file 2 [file iovs-67-10-12_s001.docx]

**Supplementary Information for**

**Annexin A1 as a key modulator of inflammatory, glial, and angiogenic signaling pathways in diabetic retinopathy**

Rafael André da Silva^1*^, Luiz Philipe de Souza Ferreira^2^, Vinícius Moraes de Paiva Roda^3^, Diego Dias dos Santos^2^, Caroline Silvério Faria^4^, [Barbara Dalmaso](https://pubmed.ncbi.nlm.nih.gov/?term=Dalmaso+B&cauthor_id=38542059) ^5^, Giovanna Serra da Silva^5^, Carolina Beltrame Del Debbio^5^, Daniel Rodrigues de Bastos^6^, Silvana Sandri^7^, Sandra Helena Poliselli Farsky^7^, Caio Vinicius Saito Regatieri^8^, and Cristiane Damas Gil.^1,2*^

***Correspondence to:** Rafael André da Silva (rasbiomedicina@gmail.com)

****Correspondence to:** Cristiane D. Gil ([cristiane.gil@unifesp.br](mailto:cristiane.gil@unifesp.br))

**This file includes:**

Supplementary Figures S1 and S2

Supporting figures (with legends)

Supplementary Figure S1

**Figure S1. Absence of changes in inflammatory markers in CNV induction.** Levels of cytokines and chemokines in retinal homogenates: (A) IL-1β, (B) IL-6, (C) IL-17, (D) CXCL1, (E) IL-2, (F) MCP-1, (G) IL-10, (H) TNF-α, and (I) IL-4. Data are expressed as mean ± SEM, with dots representing individual animals (n = 4–7 animals/group). Statistical analysis was carried out using two-way ANOVA followed by Tukey’s post hoc test.

Supplementary Figure S2

**Figure S2. Endothelial dysfunction and exacerbated angiogenesis in AnxA1^-/-^ animals induced to CNV.** Levels of growth and angiogenic factors in retinal homogenates: (A) EGF, (B) Endothelin 1, (C) FGF-2, (D) VEGF-A, (E) VEGF-C, (F) VEGF-D, (G) TGFβ-1, (H) TGFβ-2 and (I) TGFβ-3. Data are expressed as mean ± SEM, with dots representing individual animals (n = 4–7 animals/group). Statistical analysis was carried out using two-way ANOVA followed by Tukey’s post hoc test.
